# Extracellular vesicles derived from irradiated tumor cells foster immunosuppressive macrophages formation to promote esophageal squamous cell carcinoma immune evasion

**DOI:** 10.7150/ijbs.123646

**Published:** 2026-01-01

**Authors:** Shanshan Jiang, Yechun Pang, Yue Zhou, Jianjiao Ni, Li Chu, Xiao Chu, Jianghong Zhang, Yan Pan, Yida Li, Ruiting Ye, Hongru Chen, Silai Yu, Tiantian Guo, Chunlin Shao, Xi Yang, Zhengfei Zhu

**Affiliations:** 1 Department of Radiation Oncology, Fudan University Shanghai Cancer Center, Shanghai 200032, China.; 2 Department of Oncology, Shanghai Medical College, Fudan University, Shanghai 200032, China.; 3 Shanghai Clinical Research Center for Radiation Oncology, China.; 4 Shanghai Key Laboratory of Radiation Oncology, Shanghai 200032, China.; 5 Institute of Thoracic Oncology, Fudan University, Shanghai 200032, China.; 6 Department of Radiotherapy, Cancer Hospital of China Medical University, Liaoning Cancer Hospital and Institute, Cancer Hospital of Dalian University of Technology, Shenyang, 110042, Liaoning, China.; 7 Institute of Radiation Medicine, Shanghai Medical College, Fudan University, Shanghai, China.; 8 The Affiliated Suzhou Hospital of Nanjing Medical University, Suzhou Municipal Hospital, Gusu School, Nanjing Medical University, Suzhou, Jiangsu, China.

**Keywords:** esophageal squamous cell carcinoma, radiotherapy, macrophages, extracellular vesicles, programmed death ligand-1

## Abstract

**Background:** Radiotherapy (RT) remodels the tumor microenvironment (TME). Tumor-associated macrophages (TAMs) are key mediators of TME, yet how RT reprograms TAMs toward a programmed death ligand- 1(PD-L1)⁺ immunosuppressive phenotype remains unclear.

**Materials and Methods:** Esophageal squamous cell carcinoma (ESCC) subcutaneous xenografts in immunodeficient mice received localized RT or sham treatment. Tumor-infiltrating PD-L1⁺ TAMs were quantified via multiplex immunofluorescence and flow cytometry. Extracellular vesicles (EVs) derived from irradiated ESCC cells (IR-EVs) were isolated and characterized by nanoparticle tracking analysis and transmission electron microscopy. Functional assays included co-culture of IR-EVs-educated macrophages with autologous CD8⁺ T cells. RNA sequencing identified DYNLL1-AS1 as the most upregulated lncRNA in IR-EVs. Mechanistic studies employed RNA pull-down, mass spectrometry, RNA immunoprecipitation, and dual-luciferase reporter assays. Clinical validation utilized ESCC specimens for RNA *in situ* hybridization. Prognostic significance was assessed via Kaplan-Meier and Cox regression analyses.

**Results:** RT triggered ESCC cells to secrete DYNLL1-AS1-enriched EVs, which reprogrammed macrophages into PD-L1⁺ immunosuppressive TAMs. IR-EVs-educated macrophages suppressed CD8⁺ T cell proliferation and IFN-γ/ Granzyme B secretion. Mechanistically, DYNLL1-AS1 bound SEC22B, enabling its interaction with FOXP1 to activate PD-L1 transcription via promoter binding. *In vivo*, EVs carrying DYNLL1-AS1 counteract anti-PD-L1 therapy by suppressing CD8^+^ T cell function and promoting tumor growth. In ESCC patients, high DYNLL1-AS1 expression correlated with PD-L1⁺ TAM density, poor immunotherapy response, and reduced survival. Multivariate analysis confirmed DYNLL1-AS1 as an independent prognostic factor.

**Conclusions:** Radiation-induced DYNLL1-AS1 in ESCC EVs drives PD-L1⁺ TAMs immunosuppression via SEC22B/ FOXP1 signaling. Combining DYNLL1-AS1 inhibition with PD-L1 blockade may reverse RT-induced immunosuppression, offering a transformative strategy for ESCC radio-immunotherapy.

## Introduction

Radiotherapy for esophageal squamous cell carcinoma (ESCC) faces challenges with recurrence and metastasis, requiring combination therapies. Radiation-induced immunogenic cell death generates tumor antigens and partially activates antitumor immunity^1^, providing a rationale for combining radiotherapy with immunotherapy^2^. However, clinical outcomes of this combination remain suboptimal^3^. Beyond tumor-intrinsic resistance^4^, dynamic remodeling of the tumor microenvironment (TME) is a major barrier. Radiotherapy induces stromal reorganization and fibrosis^5^, activates inflammatory signaling^6^, and upregulates immune checkpoints like PD-L1^7^, collectively fostering an immune-tolerant TME. Systematic characterization and modulation of these TME dynamics are thus essential for improving ESCC radio-immunotherapy.

The TME is a complex and dynamically orchestrated ecosystem involving continuous crosstalk among malignant cells, immune cells, and stromal elements. Within the ESCC TME, membrane-bound PD-L1 expressed by malignant cells engages programmed death 1(PD-1) receptors on cytotoxic T lymphocytes, effectively inhibiting their tumoricidal capacity and facilitating immune escape. While PD-1/ PD-L1 axis blockade using monoclonal antibodies (mAbs) has demonstrated clinical efficacy across solid malignancies^8^, primary resistance and acquired immune evasion persist as major challenges in ESCC^9^. Current paradigms predominantly emphasize tumor cell-autonomous PD-L1 regulation, despite compelling evidence that PD-L1 expression by TAMs exerts superior immunosuppressive effects in specific malignancies^10^, ^11^. Within this context, TAMs exhibit context-dependent roles that can either promote or suppress tumor progression^12^. Both clinical cohorts and preclinical models confirm TAMs as dominant immune constituents within ESCC ecosystems^13^, with PD-L1^+^ TAMs subsets inducing CD8^+^ T cell exhaustion through direct ligand-receptor interactions in tumor nests and draining lymph nodes^14^. Emerging hepatobiliary carcinoma data further establish that PD-L1^high^ TAMs infiltration correlates with impaired anti-tumor immunity and reduced survival, while PD-L1 blockade partially reverses this immunosuppression^15^. Crucially, the molecular mechanisms driving radiation-induced PD-L1^+^ TAMs expansion in ESCC remain undefined, representing a critical barrier to effective radio-immunotherapy design. Furthermore, the potential contribution of PD-L1-expressing TAMs to radiation-associated immunosuppression and therapeutic resistance in ESCC has yet to be comprehensively investigated.

Extracellular vesicles (EVs) are critical mediators of inter-cellular communication and key regulators of macrophage polarization, influencing TME dynamics^16^. These nano-scale particles act as molecular couriers that transfer oncogenic cargo, including non-coding RNAs, between tumor and stromal cells to reshape the TME and drive immune evasion^17^, ^18^. Ionizing radiation dynamically alters EVs molecular composition, inducing significant modifications in RNA, protein, and lipid profiles^19^, ^20^. Radiation reprograms EVs biogenesis, secretion kinetics, and cargo sorting^21^, creating functionally dual EVs in cancer progression. For instance, glioma stem cell-derived EVs confer radiation resistance and accelerate glioblastoma progression^22^, while glioblastoma EVs enhance macrophage PD-L1 expression, amplifying RT-potentiated immunosuppression^23^. Radiation also induces ncRNA signature alterations in PBMC-derived EVs, with upregulated miR-34a-5p representing a potential biomarker for radiation exposure monitoring^24^. However, the role of EVs in mediating radiotherapy-induced crosstalk between ESCC cells and TAMs remains unelucidated.

In this study, we systematically demonstrate radiotherapy induces PD-L1^+^ TAMs polarization via EVs-transferred lncRNA DYNLL1-AS1, which scaffolds SEC22B/FOXP1 to activate PD-L1 transcription in ESCC. Clinically, DYNLL1-AS1 elevation correlates with anti-PD-L1 resistance, CD8^+^ T cell depletion, and reduced survival, redefining PD-L1 regulation from tumor- centric to an EVs-mediated intercellular paradigm. This reveals radiotherapy-educated EVs as key drivers of post-treatment immune evasion through macrophage reprogramming.

## Materials and Methods

### Cell culture

Human ESCC lines (ECA-109, KYSE-150, TE-1) and THP-1 monocytes were obtained from the Cell Bank of the Chinese Academy of Sciences (Shanghai, China). Cells were cultured under standard conditions (37°C, 5% CO₂) in RPMI-1640 medium (ECA-109, KYSE-150, TE-1) or DMEM (mEC25, mouse ESCC cells), supplemented with 10% fetal bovine serum (FBS, Gibco), 100 U/mL penicillin, and 100 μg/mL streptomycin (HyClone). For macrophage differentiation, THP-1 cells (1×10⁶ cells/mL) were treated with 100 ng/mL phorbol 12-myristate 13-acetate (PMA, Sigma-Aldrich) in RPMI-1640 for 24 hours (h). The macrophages (1×10^6^ cells/mL) were treated with EVs at 1×10^9^ particles/mL for 24 h. All cell lines underwent authentication via short tandem repeat (STR) profiling and tested negative for mycoplasma contamination.

### EVs extraction and identification

ESCC cells were irradiated (IR, 8 Gy/1F) or sham IR (NR) (X-ray, 1 Gy/min, X-RAD 320) and cultured in EVs-depleted medium. Cell culture supernatants were harvested 24 h post-irradiation for EVs isolation. EVs were isolated via sequential centrifugation (300 ×g, 10 min; 3,000 ×g, 20 min), 0.22 μm filtration, and Exosome Isolation Kit (Gefan Biotechnology). EVs size/concentration was quantified by Nanoparticle Tracking Analysis (NanoSight LM10) and morphology by transmission electron microscopy. EVs markers (CD63, TSG101) were validated via Western blot.

### EVs labeling and tracking

EVs were labeled with PKH67 (Sigma-Aldrich) following the manufacturer's protocol, incubated with macrophages at 37°C for 24 h, fixed in 4% paraformaldehyde, and counterstained with DAPI (Beyotime) for fluorescence microscopy imaging. To track DYNLL1-AS1 transfer, Cy3-labeled DYNLL1-AS1 was transfected into ESCC cells, which were co-cultured with macrophages in a Transwell system (4 μm pore, Merck Millipore) for 48 h. Cells were fixed, permeabilized with 0.01% Triton X-100, stained with DAPI, and imaged by fluorescence microscopy.

### Blockade of EVs generation by GW4869

GW4869 (Sigma-Aldrich) was dissolved in DMSO to prepare a 5 mM stock solution and diluted to 20 μM in 10% EVs-depleted FBS medium. ESCC cells were treated with GW4869 or DMSO (vehicle control) for 48 h. Culture supernatants were collected for subsequent experiments.

### RNA sequencing

Total RNA was extracted from EVs using TRIzol reagent (Invitrogen Life Technologies, USA) according to the manufacturer's instructions. LncRNA expression profiles were determined by RNA-seq (Lianchuan Biotechnology Co., LTD, China) using a HiSeq3000 (Illumina, USA).

### RNA isolation and quantitative real-time PCR (qPCR)

Total RNA was isolated from ESCC cells, ESCC cell-derived EVs, or macrophages using TRIzol (Invitrogen). Samples were lysed in 500 μL (cells/macrophages) or 200 μL (EVs) TRIzol, centrifuged (12,000 rpm, 4°C, 15 min), and processed via chloroform-isopropanol precipitation. RNA pellets were washed with 75% ethanol, air-dried, and resuspended in RNase-free water. Nuclear and cytoplasmic RNA fractions were separated using a NORGEN purification kit. RNA was reverse-transcribed with PrimeScript RT Kit (Takara), and qPCR performed using SYBR Premix Ex Taq (Takara) on a CFX96 system (Bio-Rad). Products were electrophoresed on 1.5% agarose gels stained with Gel Red (Beyotime). Primer sequences are listed in Supplementary Table 1.

### Peritoneal macrophages isolation

C57BL/6 mice (n=6 per group) received daily intravenous injections of PKH67-labeled IR-EVs, NR-EVs (1×10¹⁰ particles), or PBS for 5 days. Twenty-four hours post-final injection, peritoneal macrophages were isolated by lavage with ice-cold PBS. The lavage fluid was centrifuged at 500 × g for 10 min, and cells were plated in complete medium. After 2 h adhesion, non-adherent cells were removed by PBS washes, yielding adherent macrophages (> 95% CD11b⁺/F4/80⁺) for subsequent experiments.

### Flow cytometry assay

THP-1 derived macrophages were washed with cold PBS containing 2% BSA and resuspended in stain buffer. After Fc receptor blocking with BD Fc Block™ for 10 minutes at room temperature, cells were stained with APC anti-human CD11b, PE anti-human CD206, and BV421 anti-human CD274 antibodies for 45 minutes at 4 °C in the dark. For mouse samples, tumor single-cell suspensions were prepared using gentleMACS Dissociator and stained with PE anti-CD3, APC anti-CD8, FITC anti-CD45, PE anti-F4/80, and APC anti-CD274 antibodies. Peritoneal macrophages were similarly processed and stained with FITC anti-CD45, PE anti-F4/80, and APC anti-CD274 antibodies. All stained cells were washed twice with PBS containing 2% BSA and analyzed using a CytoFLEX flow cytometer. Data analysis was performed with FlowJo software (v7.6.1), and antibody details are provided in Supplementary Table 2.

### Enzyme-linked immunosorbent assay (ELISA)

Macrophages were treated with EVs at indicated concentrations for 48 h. Supernatants collected by centrifugation (300×g, 10 min) were analyzed for TGF-β, IL-10, and TNF-α levels using ELISA kits (MULTISCIENCES Biotech, China) per manufacturer's protocol. To evaluate T cell activation, IFN-γ and granzyme B in co-culture supernatants were quantified with eBioscience™ ELISA kits (Thermo Fisher Scientific, USA). All absorbance readings (triplicate measurements) were obtained at 450/630 nm using a microplate reader.

### Western blot analysis

ESCC cells, macrophages, and EVs were lysed in SDS buffer (Beyotime) with 1 mM PMSF, centrifuged (12,000×g, 15 min, 4 °C), and stored at -80 °C. Protein quantification used BCA Kit (Beyotime). For immunoblotting, 20 μg cell lysates/10 μg EVs proteins underwent SDS-PAGE (10% gel) and semi-dry transfer to PVDF membranes (Merck Millipore). After 90 min blocking with 5% milk/TBS-T, membranes were probed with primary antibodies (Supplementary Table 2) at 4 °C overnight, followed by HRP-secondary antibodies (1 h). ECL detection used Tanon 5200 system.

### Immunofluorescence and multiplexed immunofluorescence staining

Cells were fixed in 4% paraformaldehyde (10 min) followed by permeabilization with 0.1% Triton X-100 and blocking with 5% BSA (30 min). Primary antibodies (anti-mouse SEC22B, anti-rabbit FOXP1) were incubated overnight at 4°C, followed by 2 h room temperature incubation with secondary antibodies (anti-rabbit IgG Cy3, anti-mouse IgG FITC). Nuclei were counterstained with DAPI before mounting with anti-fade medium under coverslips for confocal imaging (Leica SP8, Germany). For multiplexed immunofluorescence, 4 μm FFPE sections underwent sequential staining with fluorochrome-conjugated primary antibodies (anti-PD-L1, anti-F4/80) and nuclear counterstaining following published protocols. Spectrally unmixed images were analyzed through IF signal-nuclear segmentation integration. Positive cells within 1-mm-diameter cylinders were quantified as mean triplicate counts (cells/spot). Antibody specifications are provided in Supplementary Table 2.

### Luciferase reporter assays

Macrophages were co-transfected with PD-L1 promoter-driven firefly luciferase reporter vectors, pRL-TK Renilla control vectors, and either DYNLL1-AS1/SEC22B expression vectors or SEC22B/FOXP1 siRNA. Dual-luciferase activities were quantified 48 h post-transfection using a commercial assay system (Genomeditech, China) with Renilla normalization.

### Transfection of lentiviral vector

The lentivirus containing DYNLL1-AS1 interference (shDYNLL1-AS1), DYNLL1-AS1 overexpression (OE-DYNLL1-AS1), negative control overexpression (OE-NC), SEC22B interference (shSEC22B) and SEC22B overexpression (OE-SEC22B) were purchased from Hanbio Biotechnology Co., LTD, China. Their negative control had random sequences. Briefly, ESCC cells or THP-1 cells were infected with the lentivirus for 24 h according to the manufacturer's instruction. When these cells could stably grow in medium containing 8 µg/mL puromycin to exclude any off-targeted cells. The efficiency of transfected in cells was monitored by qPCR or western blot assay. Following the establishment of ESCC cell lines stably overexpressing either DYNLL1-AS1 or a negative control (NC), EVs (OE-AS1 EVs and OE-NC EVs, respectively) were subsequently isolated from their culture supernatants.

### Plasmid and transfection

Using full-length SEC22B amplicons as templates, a series of SEC22B-truncated (SEC22B D1: delete 134-194 amino acids; SEC22B D2: delete 6-119 amino acids) was amplified by PCR and cloned into Flag tagged destination vectors (Genechem, China). All transfection experiments applied Lipofiter 3.0 reagents (Hanbio Biotechnology Co., LTD, China) according to the manufacturer's protocols.

### RNA pulldown assay and mass spectrum

Biotin-labeled full-length DYNLL1-AS1 and antisense DYNLL1-AS1 were synthesized *in vitro* (Yingbio Technology, Co., Ltd., China). Then the sequences were incubated with THP-1 cell lysates at room temperature for 4 h, and then the biotin labeled DYNLL1-AS1 with their binding protein partner were pulled down by streptavidin magnetic beads (ThermoFisher, USA) at 4 °C overnight. Samples were mixed with 5 × SDS loading buffer, denatured at 95 °C for 10 min, and separated by electrophoresis. The gel was fixed and treated with silver staining for color development. The different bands between sense and antisense of DYNLL1-AS1 was identified using mass spectrometry (MS) and retrieved in human proteomic library. The MS identification of proteins pulled down by DYNLL1-AS1 are listed in Supplementary Table 3.

### RNA binding protein immunoprecipitation (RIP) assay

DYNLL1-AS1 binding protein SEC22B immunoprecipitation assay was carried out using a PureBinding®RNA Immunoprecipitation Kit (Geenseed Biotech Co., Ltd., China) according to manufacturer's protocol. Briefly, 1x10^7^ cells were collected and lysate on ice in EP tubes. Protein A/G magnetic beads were resuspended and washed three times with Wash Buffer. Next, primary antibody of anti-Flag antibody (Proteintech Group, China) or anti-rabbit IgG (Cell signaling Technology, USA) was mixed with magnetic beads for 2 h at 4 ◦C. The magnetic beads with the coupled antibodies were then incubated with the lysis mixture at 4°C for 6 h, and the RNA was eluted, and qPCR was performed.

### Immunoprecipitation (IP) assay and mass spectrum

According to the manufacturer's instruction, the whole cell lysates were collected and centrifuged at 10,000 ×g for 10 min at 4 °C (Beyotime Biotechnology, China). Then 1 mL supernatant was incubated with 2 μg anti-mouse SEC22B antibody and anti-IgG antibody (mouse/rabbit) for 16 h followed by addition of 40 μL fresh protein A/G plus agarose beads (Santa Cruz Biotechnology, China) and incubated overnight at 4 °C. Purified protein complex was digested with trypsin (Gibco, USA) at 37 °C overnight to obtain the whole peptide sample and analyzed using a Q Exactive plus mass spectrometer coupled with an Easy nLC (Thermo Fisher Scientific, MA, USA). Protein was considered as positively identified if peptide score of specific peptides reached the significance threshold FDR = 0.01. The MS identification of proteins pulled down by SEC22B was listed in the Supplementary Table 4.

### Patient samples and ethical statement

This study was approved by the Ethics Committee of the Fudan University Shanghai Cancer Center, Shanghai, China (no.0504323-4-2307E). Peripheral blood samples were prospectively collected from 23 treatment-naïve stage II-III ESCC patients received radiotherapy, excluding individuals with prior immunotherapy, autoimmune disorders, or hematologic malignancies. Sequential samples were obtained ≤ 72 h pre-radiotherapy and 5-7 days post-treatment (60-66 Gy in 30-33 fractions) using BD Vacutainer® CPT™ tubes with sodium citrate. Plasma isolation involved immediate two-step centrifugation (400 × g, 15 min; 2,000 × g, 20 min, 4 °C), with aliquots (500 μL) flash-frozen in liquid nitrogen and stored at -80 °C.

### Isolation of human CD3^+^ T cells

Blood samples from ESCC patients were stored in Lymphocyte Separation Tube for Human Peripheral Blood containing sodium heparin. The supernatant was separated via centrifugation at 800×g for 15 min at 20 °C. Then the plasma of the patients was isolated for qPCR detection of DYNLL1-AS1.

PBMCs were isolated by density gradient centrifugation using Human Lymphocyte Separation Tube (DAKEWE Biological Engineering Co., LTD, China). CD3^+^ T cells from PBMCs were purified by magnetic beads (MojoSort™ Human CD3 T Cell Isolation Kit, BioLegend, USA).

### Co-cultures of macrophage and T cell systems

In a 5-day incubation, bead-purified peripheral CD3^+^ T cells were labelled with carboxyfluorescein succinimidyl ester (CFSE) and co-cultured with macrophages pretreated with ESCC-EVs at a 20:1 ratio in RPMI 1640 medium containing anti-CD3 (2 μg/mL), anti-CD28 (1 μg/mL) antibodies, and rhIL-2 (20 IU/mL). In another co-culture system, macrophages were incubated with EVs derived from ESCC cells overexpressed DYNLL1-AS1 (OE-AS1 EVs) for 24 h. CFSE-labelled CD3^+^ T cells were co-cultured with the above EVs-treated macrophages at a 20:1 ratio in a similar condition as above. After 5-day incubation, the cells were harvested for flow cytometry analysis and the supernatants were harvested for ELISA assay.

### *In situ* hybridization (ISH) and Immunohistochemistry (IHC)

A matched esophageal cancer and adjacent normal tissue microarray (TMA) were hybridized with a specific biotin-labeled DYNLL1-AS1 probe (Biotin-TTGAACCTCATTTTCTTCATCTCTCCAGACAGCTGGGTGG) (Nuohe New Biotechnology Co., Ltd., Beijing, China). All procedures were performed by strictly following the manufacturer's instructions. DYNLL1-AS1 was identified as low expression (scored ≤ 2) and high expression (scored > 3). IHC staining was conducted using streptavidin-biotin-peroxidase complex method. Briefly, ESCC tissue samples were fixed, paraffin-embedded, dewaxed, rehydrated, and antigen retrieval. Then samples were stained with CD68, PD-L1, F4/80 and CD8 antibody at 4 ℃ overnight, followed by incubation in secondary biotinylated antibody for 50 minutes at 37 ℃, and finally visualized with DAB solution and counterstained with hematoxylin. IHC stainings were examined with microscopy. The detail information of relevant antibodies was listed in Supplementary Table 2.

### Xenograft tumor mouse model

All animal procedures were conducted in strict compliance with AAALAC International guidelines under approved protocols at the Institutional Animal Care and Use Committee of Fudan University Shanghai Cancer Center SPF facility. Age-matched male BALB/c nude mice (n = 5 per group, 4-week-old) and C57BL/6 mice (n = 5 per group, 6-week-old) from SPF Biotechnology Co. (Suzhou, China) were acclimatized for 7 days under controlled conditions (22±1 °C, 55±5% humidity, 12 h light/dark cycle) with ad libitum access to autoclaved feed and water. For xenograft modeling, 4×10⁶ ECA-109 human ESCC cells suspended in 100 μL Matrigel®-PBS (1:1 v/v) were implanted into the right flank of nude mice, while mEC25 murine ESCC cells were similarly engrafted in C57BL/6 mice. Then the mice were treated with PD-L1 blocking antibodies (Bio X Cell; catalog number: BE0101, clone 10F.9G2, 10 mg/kg, i.p) or isotype control IgG (Bio X Cell; catalog number: BE0090, 10 mg/kg, i.p).

The effects of different EVs (IR-EVs, NR-EVs, OE-AS1 EVs, and OE-NC EVs) on the TME were assessed in mice randomized into experimental groups (n = 5 per group). When tumor size reached about 50 mm^3^, 10 μg of EVs (in 20 μl PBS) from different groups or 20 μl of vehicle was injected into the tumor once daily for 3 days by continuous daily injection at 9, 12, and 15 days post implant. Tumor growth was monitored daily using digital calipers, with randomization initiated when volumes reached 50±10 mm³ (calculated as (L×W²)/2, where L = long axis, W = short axis). Localized irradiation was administered via X-RAD 320ix (Precision X-Ray) using collimated fields targeting the tumor bed, with whole-body shielding via 5-mm lead plates. Terminal endpoints were predefined as tumor volume >1,500 mm³ or 14 days post-irradiation. Tumors were excised and subjected to systematic sampling. To this end, central cross-sections were fixed in formalin for subsequent multiplex IHC analysis of F4/80 and PD-L1. In parallel, fresh tumor specimens were dissociated into single-cell suspensions to enable high-dimensional profiling of the immune infiltrate using the BD FACSymphony™ flow cytometer.

### Statistical analysis

For bar graphs, data are presented as mean ± standard deviation (SD), with individual datapoints displayed. For all *in vitro* experiments, at least three independent replicates were performed. The data shown are from a single representative experiment. Intergroup comparisons were performed using parametric tests under validated normality (Shapiro-Wilk test) and homogeneity of variance (Levene's test) assumptions: independent two-tailed Student's t-tests for unpaired comparisons, paired t-tests for matched longitudinal data, and one-way ANOVA for multi-group analyses. Immunohistochemical quantification was conducted through standardized optical density measurements using ImageJ software (NIH v1.53), normalized against adjacent normal tissue baselines. Associations between DYNLL1-AS1 expression levels (dichotomized as high/low based on median cutoff) and clinicopathological parameters were evaluated via Pearson's chi-square test. Survival outcomes were analyzed using Kaplan-Meier estimators with right-censoring for loss-to-follow-up, and between-curve differences assessed via Mantel-Cox log-rank test. Prognostic predictors were identified through univariate Cox proportional hazards regression followed by multivariate adjustment for TNM stage, age, and treatment response, with proportional hazards assumptions verified through Schoenfeld residual analysis. All tests employed two-tailed thresholds (α = 0.05) without multiplicity adjustment unless specified, executed in SPSS Statistics (v22.0, IBM) and GraphPad Prism (v9.0).

## Results

### Radiation reprograms the ESCC tumor-infiltrating macrophages toward immunosuppressive phenotype

**Figure 1:**
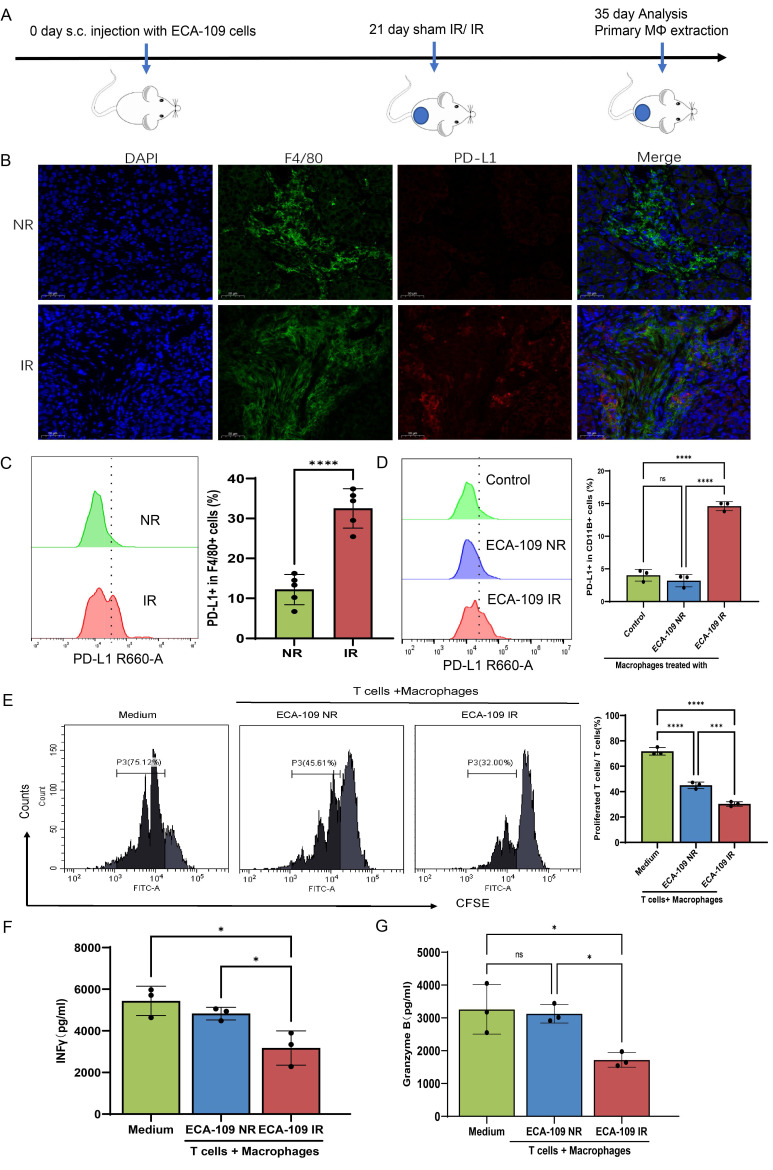
** Radiation reprograms the ESCC tumor-infiltrating macrophage toward immunosuppressive phenotype. A**, In order to assess the impact of radiation on the phenotypic changes of TAMs, subcutaneous xenograft models were established in Balb/c nude mice by injecting 4×10⁶ ECA-109 cells. Mice received local tumor irradiation (15 Gy) or sham irradiation, and tumors were harvested for analysis 35 days post-implantation (n=5 per group). **B**, **C,** Multiplex immunofluorescence (**B**) and flow cytometry analysis (**C**) of PD-L1 expression on F4/80⁺ tumor-associated macrophages (TAMs) in ESCC tissues. **D**, PD-L1 protein levels on THP-1-derived macrophages after co-culture with irradiated (IR) or non-irradiated (NR) ECA-109 cells, measured by flow cytometry. E, Proliferation of CFSE-labeled T cells after co-culture with the aforementioned macrophages, assessed by flow cytometry. **F**, **G**, ELISA detected T cell production of INF γ (**F**) and Granzyme B (**G**) after co-cultured with macrophages pretreated with IR or NR ECA-109 cells. Scale bars = 50 µm. Data are shown as the means ± SD (error bar) of at least three independent experiments. IR, irradiation, NR, sham IR, ns, no significance, * p < 0.05, ** p < 0.01, *** p < 0.001, ****p < 0.0001.

We initially examined whether radiation promotes an immunosuppressive or immunostimulatory phenotype in the ESCC TME. For this purpose, ECA-109 cells were subcutaneously implanted into Balb/c nude mice to establish xenograft models. At 21 days post-inoculation, mice received either localized irradiation or sham irradiation. A single high dose of 15 Gy was selected based on preclinical regimens validated in prior studies^25^ to achieve significant tumor cell death and robustly simulate the subsequent release of EVs and damage-associated signals. Histopathological analysis performed 14 days after irradiation revealed a marked increase in PD-L1⁺ TAMs in irradiated tumors compared with controls (**Figure 1A-C, Figure S1A**). Parallel mechanistic studies utilizing THP-1-derived macrophages co-cultured with dose-escalated ESCC cells (0-10 Gy) demonstrated radiation-induced TAMs polarization through dose-dependent upregulation of PD-L1 and CD206, peaking at 8 Gy (**Figure S1B-E**). IFN-γ (interferon-gamma) and Granzyme B serve as critical functional markers for evaluating T cell cytotoxic activity and effector functions, reflecting their capacity for immune activation and target cell elimination^26^, ^27^. We pre-treated macrophages for 24 h, and then the macrophages pre-treated with irradiated and non-irradiated ECA-109 cells were co-cultured with CD3^+^ T cells purified from the peripheral blood of patients with ESCC (**Figure S1F**). Results showed that macrophages treated by irradiated ESCC cells exhibited potent suppression of T cell proliferation (CFSE dilution: 23.4% vs 42.1%, p < 0.01) (**Figure 1E**) and effector function (IFN-γ: 3323±801 vs 4725±362 pg/ml; Granzyme B: 1771±209 vs 3145±215 pg/ml, p < 0.05) compared with sham treatment (**Figure 1F, 1G**), establishing a direct causal link between radiation-primed TAMs and T cell dysfunction. These orthogonal approaches conclusively demonstrate that radiotherapy drives ESCC immunosuppression through coordinated spatial expansion of PD-L1⁺ TAMs and functional impairment of antitumor T cell immunity.

### Radiation-induced EVs drive macrophages immunosuppression

**Figure 2:**
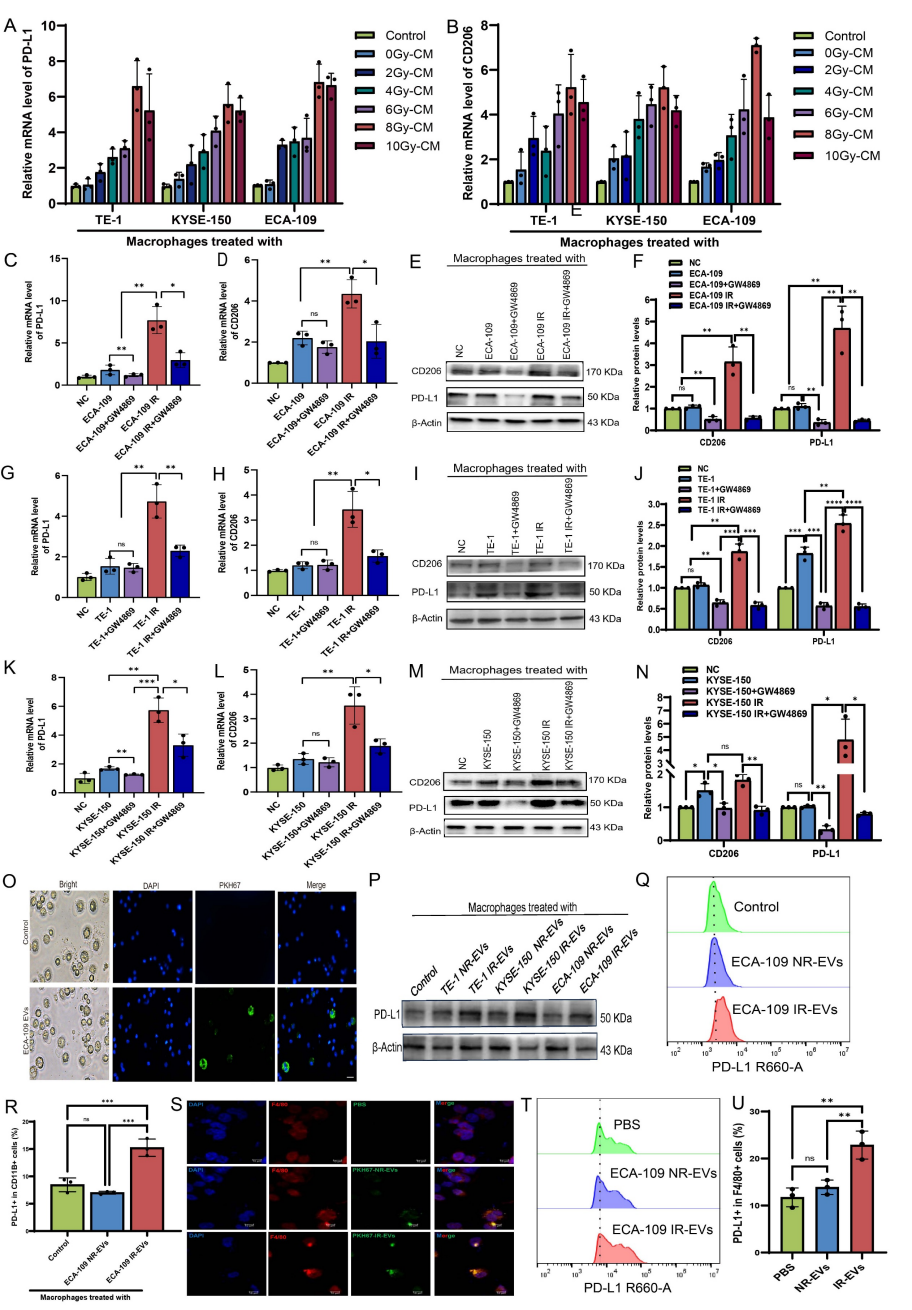
** Radiation- induced EVs drive macrophage immunosuppression. A, B,** Macrophages were co-cultured with irradiated and non-irradiated ESCC cells conditioned medium (CM) for 24h. qPCR was performed to detect the expression of PD-L1 (**A**) and CD206 (**B**) in macrophages. **C, D,** qPCR analysis was performed to detect the expression of PD-L1 (**C**) and CD206 (**D**) in macrophages treated with ECA-109 CM or CM depleted of EVs by GW4869 (an inhibitor of EVs secretion). **E, F,** Western blot analysis was used to detect PD-L1 and CD206 protein levels in macrophages treated with ECA-109 CM or CM depleted of EVs by GW4869. **G, H,** qPCR analysis was performed to detect the expression of PD-L1** (G)** and CD206 **(H)** in macrophages treated with TE-1 CM or CM depleted of EVs by GW4869. **I, J,** Western blot analysis was used to detect PD-L1 and CD206 protein levels in macrophages treated with TE-1 CM or CM depleted of EVs by GW4869. **K, L,** qPCR analysis was performed to detect the expression of PD-L1 (**K**) and CD206 (**L**) in macrophages treated with KYSE-150 CM or CM depleted of EVs by GW4869. **M, N,** Western blot analysis was used to detect PD-L1 and CD206 protein levels in macrophages treated with KYSE-150 CM or CM depleted of EVs by GW4869. **O,** Immunofluorescence was used to detect the internalization of PKH67-labeled EVs derived from ECA-109 cells by macrophages. **P,** Western blot analysis was used to detect PD-L1 protein levels in macrophages treated with IR-EVs or NR-EVs for 24 h. **Q, R,** Protein levels of PD-L1 in macrophages treated with EVs for 24 h were determined by flow cytometry. **S,** The internalization of PKH67-labeled EVs by peritoneal macrophages of mice treated with PBS, NR-EVs, and IR-EVs was detected by confocal microscopy. **T, U,** Flow cytometric analysis the expression of PD-L1 in peritoneal macrophages isolated from mice treated with PBS, NR-EVs, and IR-EVs. Scale bars= 75 μm. IR-EVs, EVs derived from irradiated ESCC cells, NR-EVs, EVs derived from sham irradiated ESCC cells, ns, no significance, * p < 0.05, ** p < 0.01, *** p < 0.001, ****p < 0.0001.

EVs are critical mediators of tumor cell-macrophage crosstalk^28^. To investigate whether irradiated ESCC cell-derived EVs modulate TAMs, conditioned medium from 8 Gy-irradiated ESCC cells was co-cultured with macrophages. This treatment markedly upregulated PD-L1 and CD206 expression (**Figure 2A, B**), whereas pharmacological depletion of EVs from ECA-109, TE-1, and KYSE-150 cell supernatants attenuated these effects (**Figure 2C-N**). EVs isolated from irradiated (IR-EVs) and non-irradiated (NR-EVs) ESCC cells were validated by transmission electron microscopy, nanoparticle tracking analysis, and Western blot for canonical EVs markers (**Figure S2A-C**). PKH67-labeled EVs were efficiently internalized by macrophages *in vitro* (**Figure 2O**). Mirroring co-culture results, IR-EVs polarized macrophages toward an immunosuppressive phenotype characterized by elevated PD-L1 expression (**Figure 2P-R**) and skewed M2/M1 marker ratios across all ESCC lines (**Figure S2D-O**). We further administered PKH67-labeled EVs from irradiated ESCC cells or non- irradiated ESCC cells to mice via tail vein injection once every 2 days for 5 times (**Figure S2P**). Then peritoneal macrophages were extracted from mice (**Figure S2Q**). We found that peritoneal macrophages derived from IR-EVs-treated mice exhibited enhanced EVs uptake (**Figure 2S**) and upregulated PD-L1 expression compared to controls (**Figure 2T, 2U, Figure S2R**). These findings collectively demonstrate that radiation-primed ESCC EVs reprogram macrophages toward an immunosuppressive state through PD-L1 induction.

### EVs derived from irradiated ESCC cells endow macrophages immunosuppressive activity against T cell-mediated anti-immunity *in vivo* and *in vitro*

**Figure 3:**
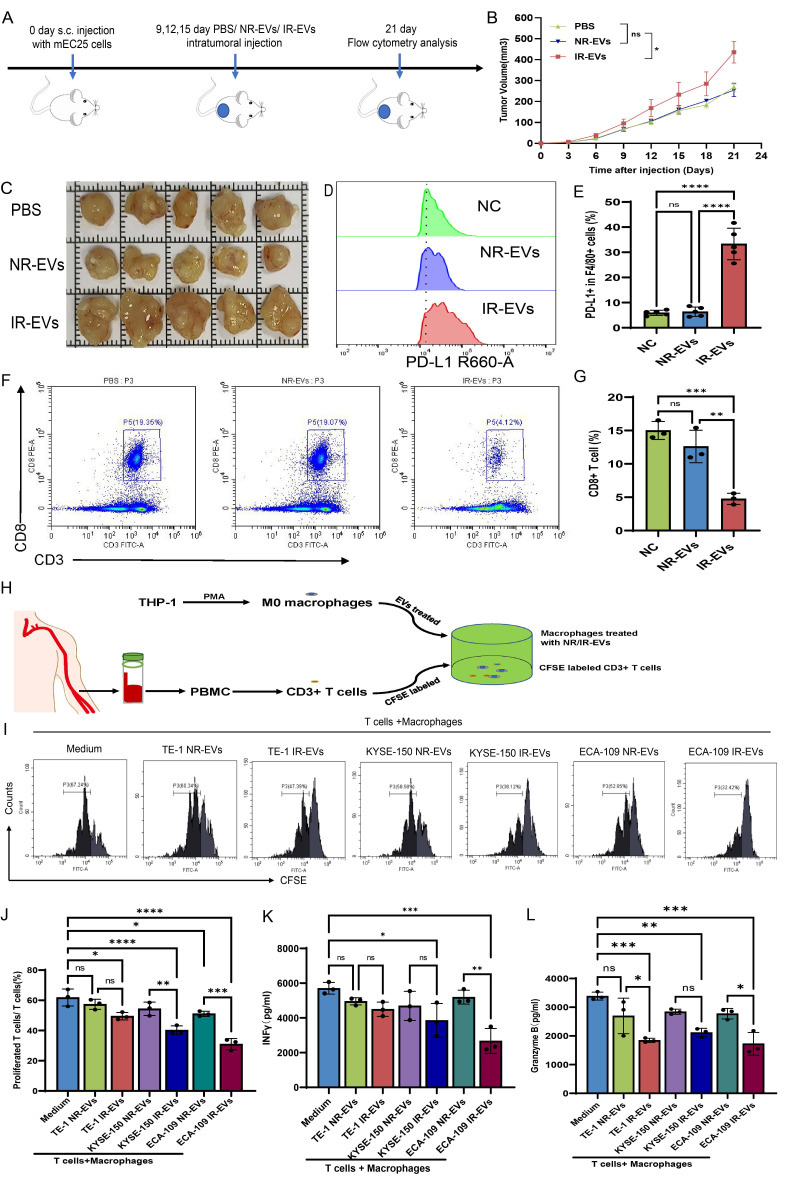
** EVs derived from irradiated ESCC cells up-regulate PD-L1 of macrophages to suppress T cells immunity *in vitro* and *in vivo*. A**, Flow chart of the experimental design *in vivo*. In a subcutaneous xenograft model established using mEC25 cells in C57BL/6 mice, PBS, NR-EVs and IR-EVs were intratumorally injected on days 9, 12, and 15, with tumors collected for analysis on day 21 (n = 5 per group). **B,** Tumor growth curves for mice in the indicated groups (n= 5 per group). **C,** The images of xenograft tumors in the indicated group of mice treated with PBS, IR-EVs or NR-EVs. **D, E,** Flow cytometry assay PD-L1 expression in macrophages *in vivo***. F, G,** Flow cytometry assay the expression of CD3^+^CD8^+^ T cells in the tumor tissue *in vivo***. H,** Flow chart depicting the experimental design *in vitro*. Peripheral CD3^+^ T cells of patients with ESCC were labeled by CFSE. THP-1 derived -macrophages were pretreated with IR-EVs and NR-EVs. Then the CD3^+^ T cells were co-cultured with macrophages for 24 h.** I, J,** Flow cytometry was performed to detect CD3^+^ T cells proliferation after co-cultured with macrophages pretreated with EVs. **K, L,** ELISA of IFN-γ (**K**) and Granzyme B (**L**) in peripheral CD3^+^ T cells after co-cultured with macrophages treated with IR-EVs and NR-EVs. Data depict the mean ± SD and are representative of three independent experiments. IR-EVs, EVs derived from irradiated ESCC cells, NR-EVs, EVs derived from sham irradiated ESCC cells, ns, no significance, *p < 0.05, **p < 0.01, ***p < 0.001, ****p < 0.0001.

To investigate whether immunosuppressive macrophages induced by IR-EVs can suppress T-cell immunity in TME, we firstly constructed a xenograft model of C57BL/6 mice by subcutaneously injected of mouse mEC25 cells. EVs derived from irradiated and non-irradiated ESCC cells were injected intratumorally on days 9, 12, 15 post-implantation. Tumor size was measured every other 2 days. The tumors were excised and analysis on days 21 post implantation (**Figure 3A**). Results showed that volumes of tumors were higher in mice that were treated with IR-EVs (**Figure 3B, C**). When compared with control and NR-EVs, IR-EVs significantly upregulated PD-L1 expression in macrophages of tumor tissues (**Figure 3D, 3E, Figure S2S**), and decreased CD8^+^ T cells levels (**Figure 3F, 3G**). Additionally, we pre-treated macrophages for 24 h, and then the macrophages pre-treated with EVs derived from irradiated and non-irradiated ECA-109 cells were co-cultured with CD3^+^ T cells purified from the peripheral blood of patients with ESCC (**Figure 3H**). Results showed that the macrophages treated by irradiated ESCC cell-derived EVs significantly inhibited T cells proliferation and effector functions (**Figure 3I-L**). Taken together, all these findings reveal that EVs derived from irradiated ESCC cells endow macrophages immunosuppressive activity against T cell-mediated anti-immunity *in vivo* and *in vitro*.

### DYNLL1-AS1 enriched in EVs derived from irradiated ESCC cells fosters immunosuppressive macrophage formation

**Figure 4:**
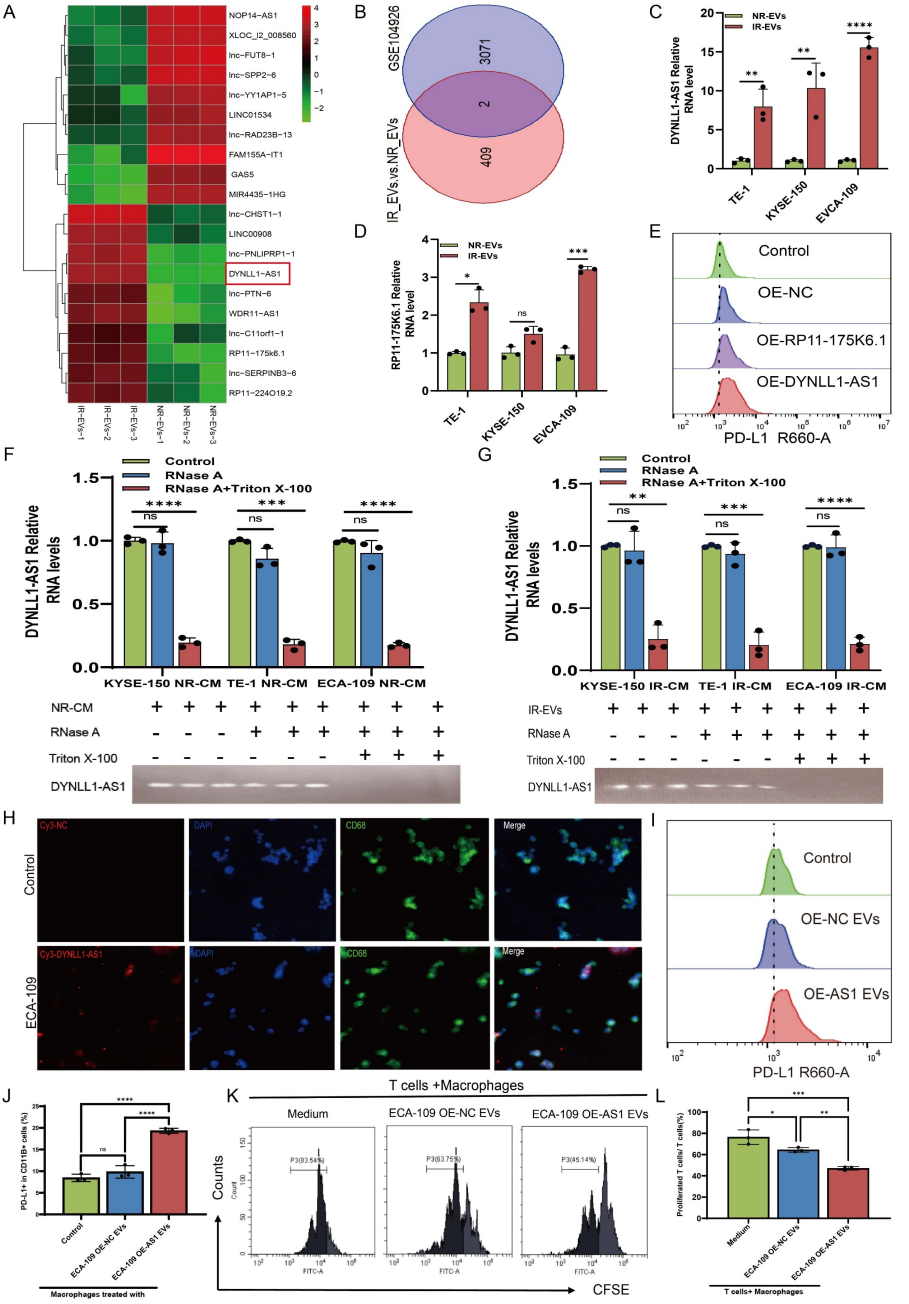
** DYNLL1-AS1 enriched in EVs derived from irradiated ESCC cells foster immunosuppressive macrophages formation. A**, LncRNAs sequencing of NR-EVs and IR-EVs are presented in a heatmap. **B,** Venn diagram of the co-expressed lncRNAs among RNA-seq of EVs and RNA-seq of plasma exosomes identifies lncRNA profiles in early-stage ESCC based on GEO database. **C,** qPCR detected DYNLL1-AS1 levels in IR-EVs and NR-EVs. **D,** qPCR detected RP11-175K6.1 levels in IR-EVs and NR-EVs. **E,** Flow cytometry was performed to detect PD-L1 expression of macrophages transfected with RP11-175K6.1 and DYNLL1-AS1 RNA.** F, G,** qPCR analysis of DYNLL1-AS1 expression in CM derived from NR ESCC cells (**F**) and IR ESCC cells (**G**) treated with RNase A (2 mg/ml) alone or combined with Triton X-100 (0.1%) for 20 min. Bottom, agarose electrophoresis of PCR product. Top, qPCR result. **H,** ESCC cells transiently transfected with Cy3-tagged DYNLL1-AS1 (Cy3-DYNL1-AS1) were co-cultured with macrophages for 24 h. Fluorescence microscopy was used to detect the red fluorescent signals in macrophages. Scale bars= 50μm. **I, J,** Flow cytometry detected PD-L1 protein in macrophages treated with EVs-overexpressed DYNLL1-AS1. **K, L,** Flow cytometry assay of the proliferation of CD3^+^ T cells after being co-cultured with macrophages treated with medium, OE-NC EVs and OE-AS1 EVs. Data depicts the mean ± SD and are representative of three independent experiments. ns, no significance, * p < 0.05, ** p < 0.01, *** p < 0.001, ****p < 0.0001.

Previous studies have described that EVs-mediated lncRNA transportation is an important process that occurs through signal transduction between macrophages and cancer cells^29^-^31^. Thus, in the present study, we performed a lncRNA sequencing to determine the expression profiles of lncRNAs in EVs derived from irradiated (IR-EVs) and non-irradiated (NR-EVs) ESCC cells (**Figure 4A, Figure S3A**). Cross-referencing these profiles with plasma exosomal lncRNA data from the GEO database (GSE104926), which contains sequencing data of lncRNAs in peripheral blood samples from patients with ESCC, revealed two conserved candidates: DYNLL1-AS1 and RP11-175K6.1 (**Figure 4B**). Although both candidate lncRNAs exhibited significant enrichment in IR-EVs (**Figure 4C, 4D**), functional validation revealed striking specificity: ectopic expression of DYNLL1-AS1, but not RP11-175K6.1, robustly upregulated PD-L1 surface expression in macrophages derived from THP1 (**Figure 4E**), establishing its non-redundant role in immune checkpoint regulation.

Moreover, DYNLL1-AS1 expression in the irradiated ESCC cells was upregulated compared with non-irradiated ESCC cells (**Figure S3B**), localized predominantly to the cytoplasm (**Figure S3C**), and secreted via EVs, as evidenced by RNase/Triton X-100 sensitivity assays (**Figure 4F, 4G**). Next, to determine whether DYNLL1-AS1 was transferred from ESCC cells to macrophages via EVs, macrophages were incubated with either regular supernatant or EVs-depleted supernatant from the cultures of IR or NR ESCC cells, respectively. Results showed that DYNLL1-AS1 levels were substantially reduced in macrophages that were treated by the supernatant in which EVs depleted pharmacologically (**Figure S3D, E**), when compared with those treated by regular supernatant without EVs depletion. Confocal microscopy confirmed Cy3-labeled DYNLL1-AS1 transfer from ESCC cells to macrophages (**Figure 4H**). Overexpression of DYNLL1-AS1 in ESCC cells (OE-AS1; **Figure S3F**) yielded EVs that upregulated PD-L1 (**Figure 4I, 4J, Figure S3G-I**) and M2 markers (CD206, TGFβ, IL10, YM1/2) while suppressing M1 markers (CD80, CD86, TNFα, IL-12) (**Figure S4A-I**) in macrophages. Functional co-cultures demonstrated that OE-AS1 EVs-primed macrophages potently inhibited CD3⁺ T cell proliferation (**Figure 4K, 4L**) and effector cytokine production (IFN-γ, Granzyme B; **Figure S4J, K**). Collectively, these findings establish DYNLL1-AS1 as a radiation-inducible EVs cargo that reprograms macrophages toward an immunosuppressive phenotype, enabling T cell dysfunction in the ESCC TME.

### DYNLL1-AS1 targeted SEC22B regulate PD-L1 expression in macrophages via FOXP1

**Figure 5:**
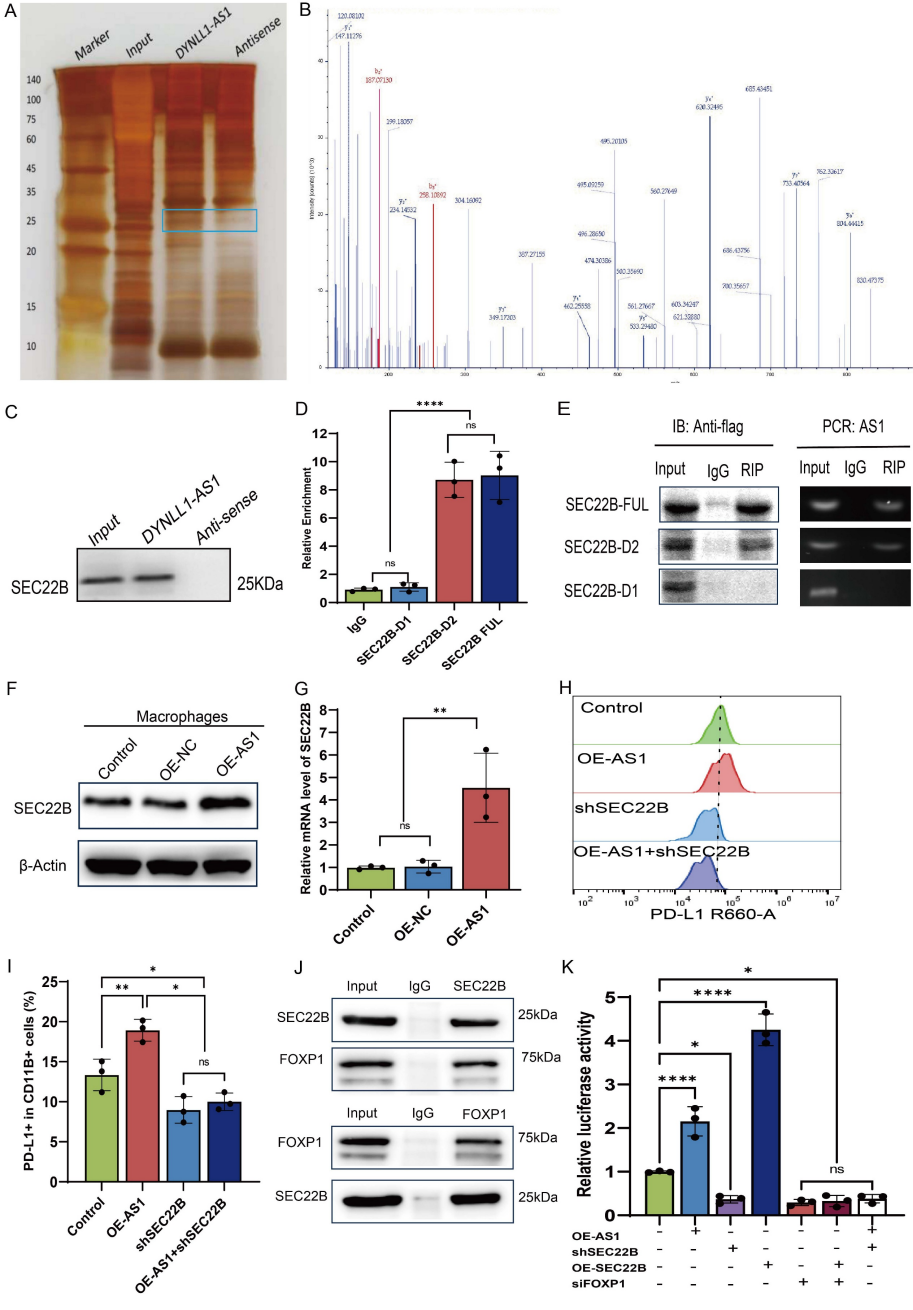
** DYNLL1-AS1 targeted SEC22B regulate PD-L1 expression in macrophages via FOXP1. A**, Protein extracted from DYNLL1-AS1 pulldown assays. **B,** Mass spectrometry identified DYNLL1-AS1-interacting protein SEC22B according to protein mass and matched unique peptides. **C,** Immunoblot analysis of the expression of SEC22B obtained from DYNLL1-AS1 pulldown assays. The antisense sequences of DYNLL1-AS1 served as negative control. **D,** RIP-PCR verified the interaction between SEC22B fragments with DYNLL1-AS1. **E,** Agarose electrophoresis of PCR product according to RIP assays with anti-flag antibodies. **F,** WB analysis the expression of SEC22B in macrophages of OE-NC and OE-DYNLL1-AS1. **G,** qPCR analysis the expression of SEC22B in macrophages of OE-NC and OE-DYNLL1-AS1. **H, I,** Flow cytometry assay PD-L1 expression in macrophages of OE-DYNLL1-AS1, shSEC22B or both OE-DYNLL1-AS1 and shSEC22B. **J,** Co-immunoprecipitation assays were performed to detect the interaction established between SEC22B and FOXP1 in macrophages using anti-SEC22B and anti-FOXP1 antibodies. **K,** The luciferase reporter activity of the PD-L1 promotor was measured in macrophages co-transfected with the corresponding reporter vector, and the overexpression vector for DYNLL1-AS1, SEC22B or downregulation vector of SEC22B or FOXP1. Data depict the mean ± SD and are representative of three independent experiments. ns, no significance, * p < 0.05, ** p < 0.01, *** p < 0.001, ****p < 0.0001.

Comprehensive molecular interrogation via RNA pulldown-mass spectrometry profiling identified SEC22B-a SNARE family vesicular trafficking protein-as the principal interactor of DYNLL1-AS1 in macrophages, with immunoblot validation confirming sequence-specific binding (**Figure 5A-C**). Structural dissection localized the interaction to SEC22B's D1 (delete 134-194 animo acids) through truncation-based RNA immunoprecipitation (RIP), establishing a critical binding interface (**Figure 5D, 5E, Figure S5A**). We then constructed OE-AS1 or knockdown SEC22B (sh-SEC22B) cell lines which was tested by qPCR (**Figure S5B, C**). Results showed that the expression of SEC22B in macrophages was positively regulated by DYNLL1-AS1 (**Figure 5F, 5G**). Functional epistasis analysis demonstrated that DYNLL1-AS1 orchestrates PD-L1 induction via SEC22B dependency, evidenced by SEC22B knockdown abolishing DYNLL1-AS1-driven PD-L1 upregulation (**Figure 5H, 5I**). In light of well-studied the mechanism by which SEC22B regulates PD-L1 expression, we employed co-immunoprecipitation (Co-IP) coupled with mass spectrometry to identify SEC22B-interacting proteins. Intersection analysis between the identified protein partners and PD-L1 transcriptional factors predicted by the JASPAR database revealed FOXP1 as a target protein (**Figure S5D**). Proteomic mapping of SEC22B interactors revealed FOXP1, a transcriptional regulator with predicted PD-L1 promoter affinity (JASPAR score >0.85) (**Figure S5E**), exhibiting significant co-expression in ESCC cohorts (GEPIA: R=0.52, p=7.3e-^15^, **Figure S5F**). Co-immunoprecipitation and subcellular localization studies confirmed direct SEC22B-FOXP1 interaction and cytoplasmic complex formation (**Figure 5J, Figure S5G**). Dual-luciferase reporter assays further demonstrated that upregulation of DYNLL1-AS1 or SEC22B enhanced PD-L1 expression in macrophages, whereas targeted knockdown of either SEC22B or FOXP1 markedly attenuated PD-L1 transcriptional activity. Furthermore, dual genetic manipulation experiments revealed distinct regulatory hierarchies: co-upregulation of DYNLL1-AS1 with SEC22B down-regulation resulted in diminished PD-L1 expression, indicating that DYNLL1-AS1 modulates PD-L1 expression through SEC22B regulation. Conversely, simultaneous SEC22B overexpression and FOXP1 knockdown attenuated PD-L1 levels, demonstrating SEC22B's dependence on FOXP1 for PD-L1 transcriptional control (**Figure 5K**). These data collectively define a tripartite axis wherein EVs-shuttled DYNLL1-AS1 engages SEC22B's D1 to license FOXP1-mediated transcriptional activation of PD-L1, thereby establishing SEC22B as a druggable linchpin connecting vesicular trafficking to regulate TAMs programming.

### DYNLL1-AS1 inhibit the efficacy of immunotherapy for ESCC *in vivo*

**Figure 6:**
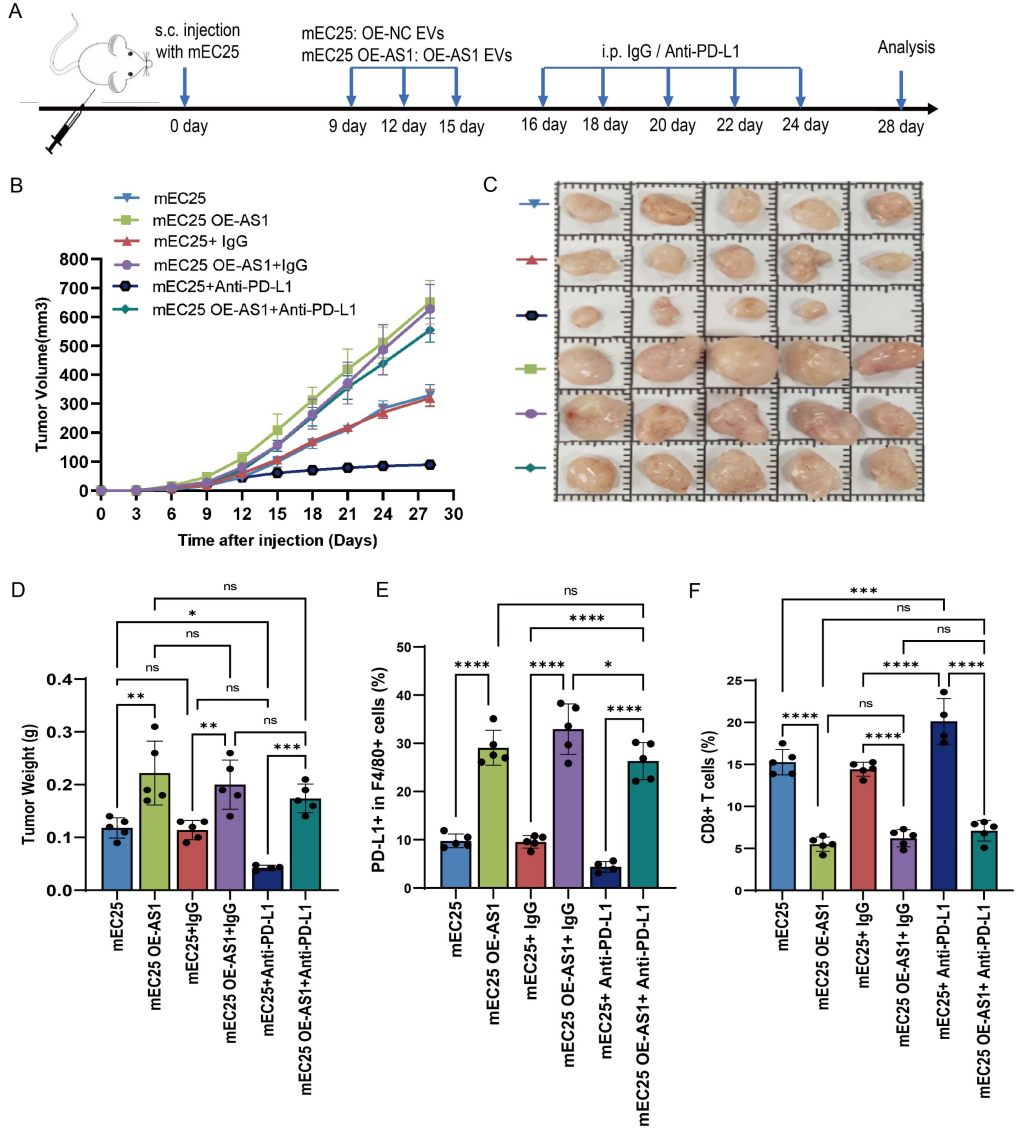
** Upregulation of DYNLL1-AS1 in cancer cells inhibit the efficacy of immunotherapy for ESCC *in vivo*. A,** Flow chart of the experimental design *in vivo*. A total of 4×10^6^ mEC25 cells were injected subcutaneously into the right flank of each C57BL/6 mouse. Tumor-bearing mice were randomized into two cohorts and treated with either OE-AS1 EVs (mEC25 OE-AS1 group) or OE-NC EVs (mEC25 group) on days 9, 12, and 15. Each group subsequently received either anti-PD-L1 blockade (10 mg/kg, intraperitoneally every 3 days) or an IgG control (n = 5 for each group). **B,** Tumor growth of the mice with different treatments. **C,** The images of xenograft tumors in the indicated group of mice treated with different treatments. **D,** Tumor weight of the mice with different treatments. **E,** Flow cytometry assay of the expression of PD-L1^+^ TAMs in the tumor tissue. **F,** Flow cytometry assay of the expression of CD3^+^CD8^+^ T cells in the tumor tissue. Data depict the mean ± SD. ns, no significance, * p < 0.05, ** p < 0.01, *** p < 0.001, ****p < 0.0001.

Next, we investigated whether DYNLL1-AS1 treatment induce immunosuppression *in vivo*. We employed an immunocompetent C57BL/6 model wherein mice bearing mEC25 ESCC tumors. The tumor-bearing mice were then randomly divided into two cohorts and treated with either OE-AS1 EVs (mEC25 OE-AS1 group) or OE-NC EVs (mEC25 group). Following EVs treatment, each group was further administered either anti-PD-L1 antibody (10 mg/kg, i.p., every 3 days) or an IgG control (**Figure 6A**). Compared with the OE-NC group, tumors of OE-AS1 group exhibited accelerated growth kinetics. While with PD-L1 inhibition significantly attenuating OE-NC group tumor progression but showing limited efficacy in OE-AS1 cohorts (**Figure 6B-D**). Flow cytometric profiling revealed OE-AS1 group tumors harbored elevated PD-L1⁺F4/80⁺ TAMs (**Figure 6E, Figure S6A**) and reduced CD3⁺CD8⁺ T cell infiltration (**Figure 6F, Figure S6B**), a phenotype corroborated by multiplex IHC showing spatial exclusion of cytotoxic lymphocytes from tumor cores (**Figure S6C**). PD-L1 blockade reversed this immunosuppressive signature, decreasing PD-L1⁺ TAMs and augmenting CD8⁺ T cell density in OE-NC tumors, while OE-AS1 tumors maintained therapeutic resistance. These *in vivo* findings mechanistically converge with our molecular data, demonstrating that EVs-encapsulated DYNLL1-AS1 orchestrates PD-L1-dependent immune evasion through dual modulation of TAMs checkpoint expression, thereby establishing a therapeutically targetable axis in radioresistant ESCC.

### DYNLL1-AS1 drives radiotherapy- induced immunosuppression via macrophage reprogramming and predicts therapeutic resistance in ESCC patients

**Figure 7:**
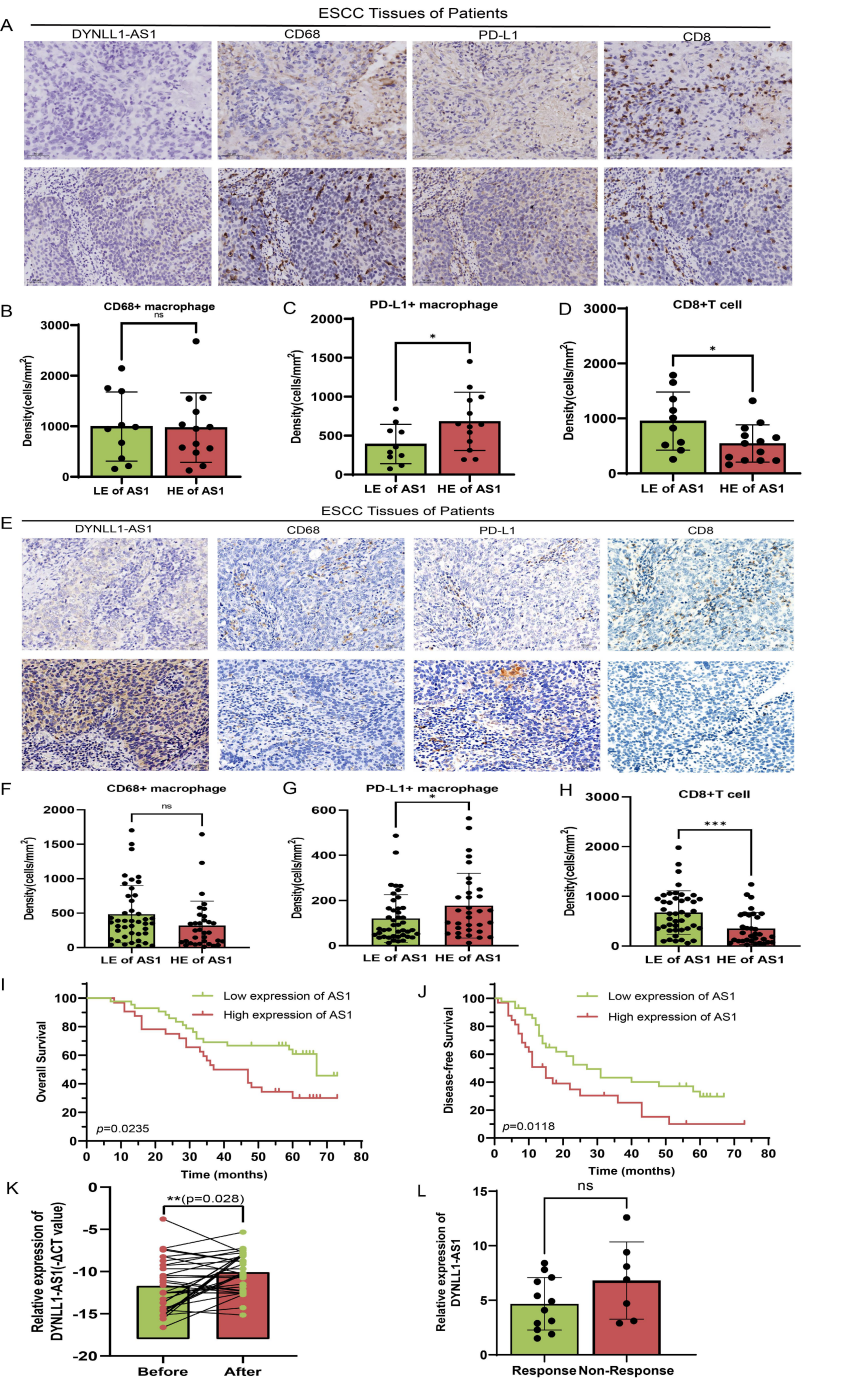
** DYNLL1-AS1 drives radiotherapy- induced immunosuppression via macrophages reprogramming and predicts therapeutic resistance in ESCC patients. A,** The expression pattern of DYNLL1-AS1, CD68, PD-L1 and CD8 in tumor tissues of ESCC patients with neoadjuvant immunotherapy measured by ISH and IHC, respectively.** B, C, D,** The relationship between DYNLL1-AS1 levels and expression of CD68 (**B**), PD-L1 (**C**) and CD8 (**D**) in tumor tissues of ESCC patients with neoadjuvant immunotherapy. **E,** The expression pattern of DYNLL1-AS1, CD68, PD-L1 and CD8 in tissues of treatment-naïve ESCC patients undergoing radical esophagectomy was measured by ISH and IHC, respectively. **F, G, H,** The relationship between DYNLL1-AS1 levels and expression of CD68 (**F**), PD-L1(**G**) and CD8 (**H**) in tumor tissues of ESCC patients initially treated by surgery. **I, J,** Kaplan-Meier analysis of overall survival rates (**I**) and disease-free survival rates (**J**) in high and low DYNLL1-AS1 expression groups in tumor tissues of ESCC patients treated with initial surgery. **K,** DYNLL1-AS1 levels were detected using real-time PCR in plasma from ESCC patients before and after radiotherapy. **L,** The relationship between DYNLL1-AS1 expression and efficacy of radiotherapy in ESCC patients after radiographic assessment at 3-month follow-up. Scale bars= 50 μm. Data are shown as the means ± SD. LE low expression, HE high expression, ns, no significance, * p < 0.05, ** p < 0.01, *** p < 0.001, ****p < 0.0001.

To investigate the clinical effects of DYNLL1-AS1 in patients with ESCC, the DYNLL1-AS1 levels were detected in the samples collected from patients with neoadjuvant immunotherapy, surgery and radiotherapy. In neoadjuvant immunotherapy recipients (Supplementary Table 5), high expression of DYNLL1-AS1 in tumors displayed attenuated CD8⁺ T cell infiltration, amplified PD-L1⁺ TAMs densities (**Figure 7A-D**), and diminished pathological response rates (tumor regression grade 3: 23.1% vs 60%, Supplementary Table 6). Subsequent analysis of DYNLL1-AS1 expression in treatment-naïve ESCC patients undergoing radical esophagectomy established its prognostic significance (Supplementary Table 7). Mechanistically, tumor microenvironment analysis confirmed that high DYNLL1-AS1 expression (ISH score >3) correlated with PD-L1⁺ TAMs enrichment and CD8⁺ T cell exclusion (**Figure 7E-H**). And the expression of DYNLL1-AS1 was significantly associated with larger tumor size, advanced T stage, higher N stage, and elevated TNM stage in patients (Supplementary Table 8). Critically, survival analytics identified DYNLL1-AS1 as an independent predictor of adverse outcomes, with high-expressing patients exhibiting reduced 5-year overall survival (OS) (34.2% vs 68.9%; hazard ratio [HR]=2.87, p=0.0235) and disease-free survival (DFS) (12.1% vs 32.4%; HR=3.12, p=0.0118;** Figure 7I, J**). Multivariate Cox regression validated its independent prognostic value for both OS (HR=0.025, 95% confidence interval [CI] 0.003-0.189; Table 1) and DFS (HR=0.057, 95% CI 0.005-0.642; Table 2). Moreover, in a prospective cohort of treatment-naïve ESCC patients receiving radiotherapy (n=23) (Supplementary Table 9), clinical interrogation of DYNLL1-AS1 pathobiology revealed its role as a radiotherapy-responsive immunomodulator. Longitudinal plasma profiling demonstrated a 2.2-fold increase in circulating DYNLL1-AS1 levels post-radiotherapy (p=0.028, paired t-test) (**Figure 7K**). Based on radiographic follow-up data at 3 months, we stratified patients into responders and non-responders. Analysis of the association between post-radiotherapy plasma DYNLL1-AS1 levels and therapeutic efficacy revealed a non-significant trend, wherein elevated DYNLL1-AS1 was associated with reduced radiotherapeutic efficacy (**Figure 7L**). These multimodal findings establish DYNLL1-AS1 as a theranostically actionable regulator of radiotherapy-induced immunosuppression through coordinated TAMs reprogramming and adaptive immune evasion, while providing a theranostic biomarker for optimizing radio-immunotherapy synergy in ESCC.

## Discussion

This study delineates a previously unrecognized axis of radiation-induced immune evasion in ESCC, revealing that radiotherapy triggers tumor-derived EVs to deliver lncRNA DYNLL1-AS1 that reprograms macrophage PD-L1 expression via SEC22B/FOXP1 signaling. These findings address critical knowledge gaps regarding radiation-mediated TME remodeling and provide mechanistic insights into the paradoxical immunomodulatory effects of radiotherapy. While prior studies have established radiation's capacity to induce PD-L1 expression in tumor cells^11^, our work fundamentally expands this paradigm by demonstrating that radiotherapy concurrently amplifies PD-L1^+^ TAMs populations through EVs-mediated horizontal gene transfer-a mechanism with profound implications for radio-immunotherapy combinations.

The TME forms a complex ecosystem composed of malignant cells and various stromal elements. Within this milieu, TAMs represent the most prevalent immune population^32^. These plastic immune cells exist along an activation spectrum from pro-inflammatory M1 to immunosuppressive M2 phenotypes^33^. Radiation therapy exerts paradoxical immunomodulatory effects, simultaneously stimulating anti-tumor immunity while fostering immunosuppressive TME remodeling^34^. Current literature reveals conflicting patterns of radiation dose-dependent macrophage polarization. Genard *et al.* describe low-dose (<2 Gy) radiation favoring M2 polarization versus high-dose (>8 Gy) promoting M1 differentiation^35^, while Meng *et al.* report M2 polarization following both fractionated (2 Gy ×10) and ablative (20 Gy) regimens^36^. Conversely, Klug *et al.* propose low-dose radiation induces M1 polarization through iNOS^+^ macrophage differentiation^37^. Our experimental data reconcile these discrepancies by demonstrating that ESCC-derived signals dominate over direct radiation effects in shaping macrophage phenotypes. Specifically, co-culture with ESCC cells irradiated at 8 Gy significantly enhanced M2 marker expression (CD206, IL10) while suppressing M1 markers (iNOS, TNF-α) in macrophages. This aligns with clinical observations that high-dose irradiation (>4 Gy) paradoxically accelerates tumor progression through early M2-TAMs recruitment and angiogenic activation^38^, ^39^. Importantly, our dose-response experiments reveal a critical threshold at 8 Gy, where radiation-primed ESCC cells acquire maximal capacity to induce pro-tumoral M2 polarization, suggesting dose optimization strategies may help mitigate radiotherapy-induced immunosuppression. Notably, the dose-dependent polarization effects observed here (8 Gy preferentially inducing M2 phenotypes) reconcile conflicting literature on radiation dosage and macrophage polarization^37^-^39^. The demonstrated dominance of high-dose radiation in fostering immunosuppressive TAMs reprogramming underscores the need for dose fractionation strategies that balance tumor control with immune preservation.

The PD-L1 checkpoint molecule serves as a critical regulator of T-cell activation through engagement with its receptor PD-1 on lymphocytes. While radiation-induced PD-L1 upregulation has been documented in both malignant cells and stromal components of the TME^40^, current understanding remains predominantly tumor cell-centric. Emerging clinical evidence positions myeloid cell-derived PD-L1 as a potent immunosuppressive mediator in various malignancies^10^, ^11^, yet its radiation-inducible expression in ESCC-associated TAMs remains unexplored. Our experimental paradigm demonstrates that radiotherapy enhances PD-L1 expression in TAMs in nude mouse xenograft models and macrophages co-cultured with irradiated ESCC cells exhibit marked PD-L1 surface elevation with subsequent functional assays confirming their capacity to suppress T-cell proliferation and effector functions. These findings align with recent glioblastoma studies showing radiotherapy-enhanced PD-L1 expression in TAMs^23^, suggesting conserved mechanisms across tumor types. Therapeutically, our data underscore the potential clinical value of combinatorial approaches targeting macrophage PD-L1 to overcome radiation-induced immune suppression.

EVs are secreted by almost all cell types and exert intercellular communication and cargo transfer functions^41^. It carries a variety of signaling molecules and provide a new avenue for facilitating cell-to cell communication^42^. These nano-vesicles regulate tumor progression through diverse molecular cargoes (proteins, mRNAs, miRNAs, lncRNAs) that modulate immune cell function. Accumulating evidence indicates that tumor-derived exosomes induce immunosuppression by targeting dendritic cells^43^, natural killer cells^44^, and myeloid-derived suppressor cells^45^. The discovery that irradiated ESCC cells secrete DYNLL1-AS1-enriched EVs to drive macrophage PD-L1 upregulation introduces three key conceptual advances. It establishes lncRNAs as critical mediators of radiation-induced intercellular communication, extending beyond canonical protein/miRNA-based EV signaling^46^-^48^. Our findings resonate with emerging evidence that non-tumor cell PD-L1 expression-particularly in myeloid populations-exerts dominant immunosuppressive effects in certain malignancies^10^, ^23^. The observation that radiation-primed EVs induce PD-L1^+^ M2-like macrophages aligns with reports of radiotherapy driving myeloid cell-mediated immune suppression in glioblastoma^23^ and pancreatic cancer^49^. Clinically, the correlation between elevated DYNLL1-AS1 levels, PD-L1^+^ TAMs infiltration, and poor immunotherapy response has immediate translational implications. Our findings extend the oncogenic repertoire of DYNLL1-AS1 beyond its reported roles in tumor proliferation to encompass immune microenvironment remodeling^50^, ^51^. The differential effects of radiation doses on macrophage polarization (8 Gy vs lower doses) suggest that conventional fractionation regimens may inadvertently foster immune resistance-a hypothesis requiring validation in clinical cohorts. Importantly, the reversibility of PD-L1 blockade resistance through DYNLL1-AS1 inhibition in preclinical models provides proof-of-concept for targeting this axis to enhance radio-immunotherapy efficacy.

We identify the SEC22B/FOXP1 axis as a novel PD-L1 regulatory pathway in TAMs, bridging vesicular trafficking machinery with transcriptional control of immune checkpoints-a mechanistic link not previously described in radiation biology. The mechanistic elucidation of DYNLL1-AS1/SEC22B/FOXP1 signaling unveils multi-layered regulation of PD-L1 in TAMs. SEC22B's dual role as both a vesicular trafficking component^52^, ^53^ and transcriptional co-regulator-through its physical interaction with FOXP1-represents a paradigm shift in understanding immune checkpoint control. This discovery complements recent work establishing STAT3-dependent regulation of PD-L1 in TAMs^54^, as our data reveal FOXP1 as a downstream effector integrating STAT3 signaling^55^ and vesicular lncRNA inputs. The identification of SEC22B's C-terminal binding domain with DYNLL1-AS1 provides structural insights into how EVs cargo can directly manipulate host cell transcriptional machinery. While this study focuses on ESCC, the identified mechanism likely has broader relevance across malignancies treated with radiotherapy. The conserved nature of SEC22B-mediated vesicular trafficking^52^ and FOXP1's established role in myeloid differentiation^56^ suggest this pathway may represent a universal axis of therapy-induced immune evasion. Future studies should explore circulating EV-DYNLL1-AS1 as a dynamic biomarker for monitoring radiotherapy-induced immune remodeling and predicting PD-L1 blockade responsiveness.

This work is subject to certain limitations. While the employed model provides a robust and standardized system for evaluating tumor growth and treatment response, it is critical to note that it fails to recapitulate the complex native TME, including the stromal interactions, immune context, and vascular architecture of orthotopic or spontaneous models. Additionally, the absence of a functional immune system in the athymic nude mouse host does not permit the assessment of any immune-mediated therapy effects. Consequently, while our results establish the treatment's direct anti-tumor efficacy, their definitive confirmation necessitates further investigation.

## Conclusions

In summary, our present study has demonstrated that DYNLL1-AS1 levels were significantly higher in irradiated ESCC cells-derived EVs, which can polarize TAMs toward an M2 phenotype and upregulate expression of PD-L1 in macrophages promoting immunosuppressive phenotype and triggered tumor immune escape mechanisms through SEC22B/FOXP1 signal pathway. Furtherly, DYNLL1-AS1, upregulated in the plasma of ESCC patients after radiotherapy and ESCC tissues, may be a prognostic marker and a therapeutic target in ESCC. However, extensive future studies are still warranted to validate these findings.

## Supplementary Material

Supplementary figures and tables.

## Figures and Tables

**Table 1:** Cox proportional-hazards regression model for OS analysis in patients with ESCC

Variables	Univariate analysis			Multivariate analysis		
	HR	95% CI	*p* value	HR	95% CI	*p* value
Age (years) (≤ 60 vs > 60)	0.413	0.161-1.058	0.065	0.39	0.090-1.680	0.206
Gender (Male vs Female)	2.109	0.587-7.577	0.253			
Tumor size (cm) (≤ 3 vs > 3)	0.768	0.309-1.909	0.507			
Tumor location						
Upper	Reference					
Middle	2.143	0.174-26.329	0.552			
Lower	0.771	0.299-1.990	0.591			
Tumor differentiation						
Well	Reference					
Modest	0.231	0.051-1.043	0.057			
Poor	0.802	0.286-2.245	0.674			
pN (N0+N1 vs N2+N3)	0.100	0.026-0.388	0.001	0.037	0.005-0.292	0.002
pT (T1+T2 vs T3+T4)	0.150	0.053-0.426	0.000	0.031	0.004-0.226	0.001
pTNM stages (I+II vs III+IV)	0.208	0.077-0.560	0.002	1.000	0.230-4.348	1.000
Adjuvant therapy (Yes vs No)	0.554	0.215-1.425	0.220			
AS1 expression (Low vs High)	0.135	0.048-0.377	0.000	0.025	0.003-0.189	0.000

Abbreviation: AS1, DYNLL1-AS1.

**Table 2:** Cox proportional-hazards regression model for DFS analysis in patients with ESCC

Variables	Univariate analysis			Multivariate analysis		
	HR	95% CI	*p* value	HR	95% CI	*p* value
Age (years) (≤ 60 vs > 60)	0.882	0.298-2.615	0.821			
Gender (Male vs Female)	1.833	0.468-7.188	0.385			
Tumor size (cm) (≤ 3 vs > 3)	0.857	0.294-2.497	0.778			
Tumor location						
Upper	Reference					
Middle	3.667	0.323-41.590	0.294			
Lower	1.069	0.343-3.331	0.908			
Tumor differentiation						
Well	Reference					
Modest	0.625	0.127-3.066	0.562			
Poor	0.609	0.168-2.207	0.451			
pN (N0+N1 vs N2+N3)	0.043	0.005-0.367	0.004	0.027	0.002-0.490	0.015
pT (T1+T2 vs T3+T4)	0.225	0.069-0.732	0.013	0.685	0.117-4.013	0.675
pTNM stages (I+II vs III+IV)	0.167	0.050-0.551	0.003	0.653	0.116-3.679	0.629
Adjuvant therapy (Yes vs No)	0.317	0.093-1.085	0.067	0.411	0052-3.232	0.398
AS1expression (Low vs High)	0.046	0.009-0.238	0.000	0.057	0.005-0.642	0.020

Abbreviation: AS1, DYNLL1-AS1.

## Abbreviations

ESCC: Esophageal squamous cell carcinoma
EVs: Extracellular vesicles
TAM: Tumor-associated macrophages
OS: Overall survival
DFS: Disease-free survival
RT: Radiotherapy
IR: Irradiated
NR: Non-Irradiated
TME: Tumor microenvironment
PD-L1: Programmed death ligand 1
RIP: RNA immunoprecipitation
ISH: *In situ* hybridization
IHC: Immunohistochemistry
TAA: Tumor-associated antigens
ncRNAs: non-coding RNAs
PBMC: Peripheral blood mononuclear cell
ELISA: Enzyme-linked immunosorbent assay
IP: Immunoprecipitation
CFSE: Carboxyfluorescein succinimidyl ester
