# Supplementary material for: Extracellular vesicles derived from irradiated tumor cells foster immunosuppressive macrophages formation to promote esophageal squamous cell carcinoma immune evasion
Source: Int J Biol Sci. 2026 Jan 1;22(2):802–22. doi: 10.7150/ijbs.123646 (PMC12781079; doi:10.7150/ijbs.123646)
Supplement: Supplementary file 1 — Supplementary figures and tables. [file ijbsv22p0802s1.pdf]

Supplemental Figures

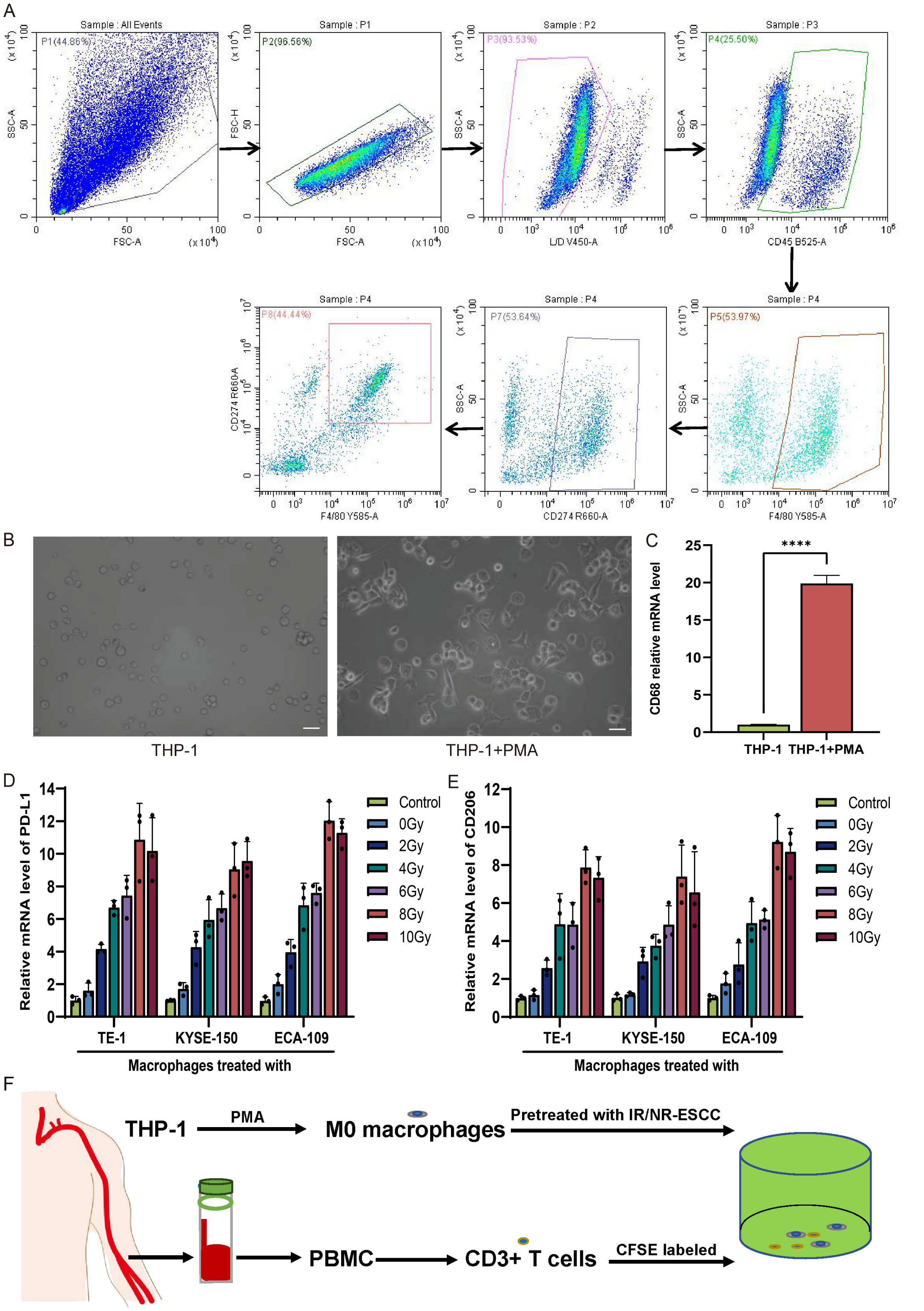

Figure S1 Radiation reprograms the ESCC tumor-infiltrating macrophages toward

immunosuppressive phenotype. A, Flow chart depicting the expression of PD-L1 in macrophages of tumor tissues from mice using flow cytometry assay. **B,** Representative image of macrophages derived from THP-1 cells treated with phorbol 12-myristate 13-acetate (PMA) for 24 h. **C,** qPCR analysis was performed to detect the expression of the macrophages marker CD68. **D, E,** Macrophages were co-cultured with IR and NR ESCC cells for 24 h. qPCR was performed to detect the expression of PD-L1 **(C)** and CD206 **(D)** in macrophages. **F,** Flow chart depicting the experimental design in vitro. Peripheral $CD3^+$ T cells of patients with ESCC were labeled by CFSE. THP-1 derived -macrophages were pretreated with IR or NR ECA-109 cells. Then the $CD3^+$ T cells were co-cultured with pretreated macrophages for 24 h. Scale bars = 50 μm. Data are shown as the means ± SD (error bar) of at least three independent experiments. IR, irradiation, NR, sham IR, **** $p < 0.0001$.

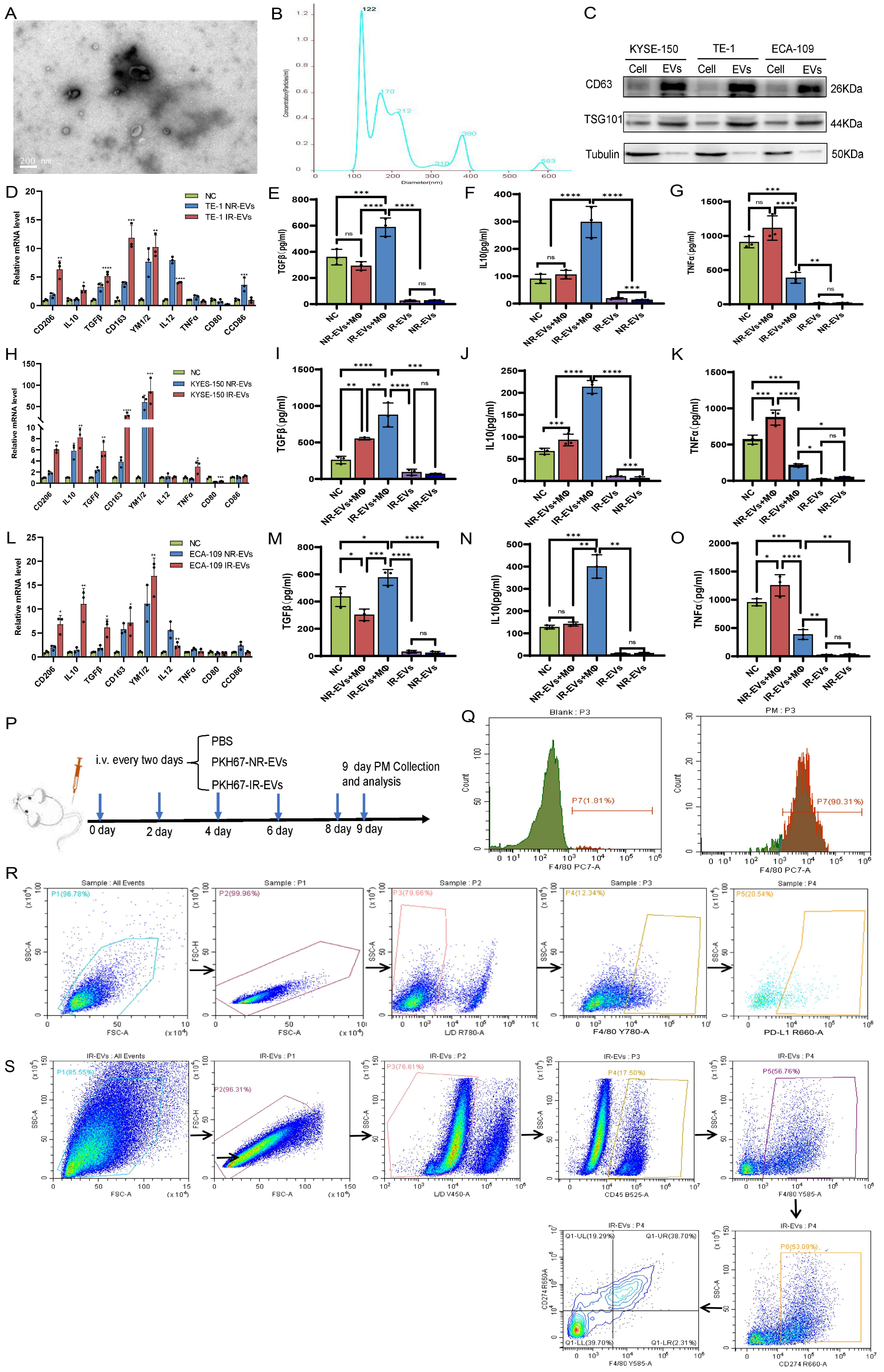

Figure S2 Radiation- induced EVs drive macrophages immunosuppression. A, Transmission

electron micrograph of ESCC cell-derived EVs. Scale bars= 200 nm. **B,** Characterization of ESCC cell-derived EVs were detected by nano-sight particle tracking analysis. **C,** Western blot analyses were performed to detect typical EVs markers CD63 and TSG101 in ESCC cells and EVs. **D, E, F, G,** qPCR analysis **(D)** and ELISA essay **(E, F, G)** were performed to detect the markers expression of M1 and M2 macrophages treated with IR-EVs and NR-EVs of TE-1 cells. **H, I, J, K,** qPCR analysis **(H)** and ELISA essay **(I, J, K)** were performed to detect the markers expression of M1 and M2 macrophages treated with IR-EVs and NR-EVs of KYES-150 cells. **L, M, N, O,** qPCR analysis **(L)** and ELISA essay **(M, N, O)** were performed to detect the markers expression of M1 and M2 macrophages treated with IR-EVs and NR-EVs of ECA-109 cells. **P,** Flow chart depicting the experimental design. PKH67-labeled EVs were injected to C57BL/6 mice intravenously through the tail vein at the same dose (1×10^{10} particles/100 μL PBS per mouse) once every 2 days (5 times). The day after the last injection, the mice were sacrificed, and peritoneal macrophages (PM) were extracted for subsequent experiments (n = 10 for each group). **Q,** Peritoneal macrophages marker F4/80 were detected by flow cytometry. **R,** Flow chart depicting the expression of PD-L1 in peritoneal macrophages of mice using flow cytometry assay. **S,** Flow chart depicting the expression of PD-L1 in TAMs of mice using flow cytometry assay. ns no significance, * $p < 0.05$, ** $p < 0.01$, *** $p < 0.001$, ****$p < 0.0001$.

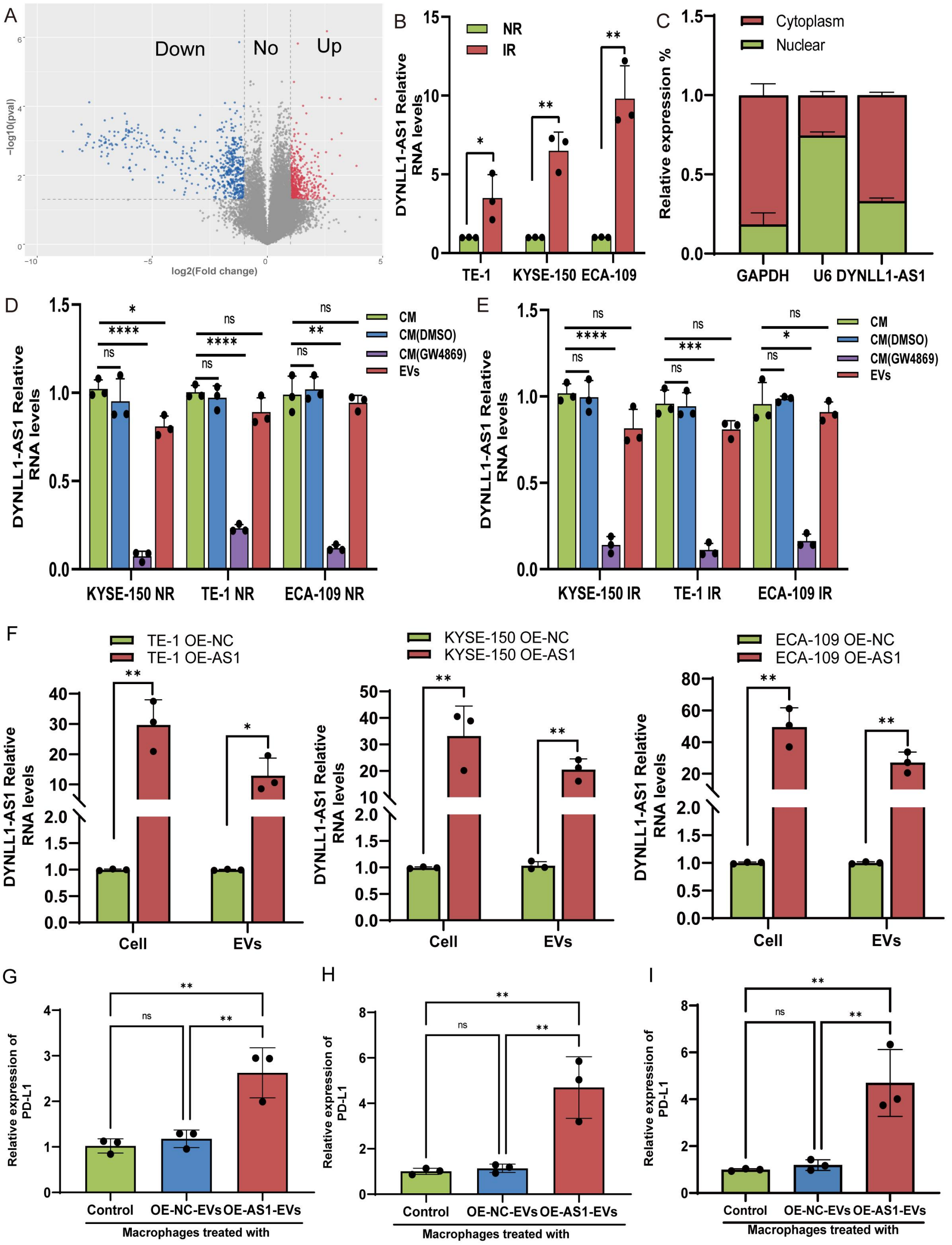

Figure S3 DYNLL1-AS1 enriched in EVs derived from irradiated ESCC cells foster immunosuppressive macrophages formation. A, Volcano plot displayed differentially expressed lncRNAs. **B,** qPCR analysis was performed to detect the expression of DYNLL1-AS1 in IR and NR ESCC cells. **C,** qPCR analysis was performed to detect the expression of DYNLL1-AS1 in cytoplasm or nuclear of ECA-109 cells. **D,** qPCR analysis was performed to detect the expression of

DYNLL1-AS1 in macrophages treated with EVs, CM or CM depleted of EVs by GW4869 of NR ESCC cells. **E,** qPCR analysis was performed to detect the expression of DYNLL1-AS1 in EVs, CM or CM depleted of EVs by GW4869 of IR ESCC cells. **F,** qPCR analysis was performed to detect the expression of DYNLL1-AS1 in ESCC cells and EVs. **G,** qPCR assay results revealed that OE-AS1-EVs derived from TE-1 cells upregulated the expression of PD-L1 in macrophages. **H,** qPCR assay results revealed that OE-AS1-EVs derived from KYSE-150 cells upregulated the expression of PD-L1 in macrophages. **I,** qPCR assay results revealed that OE-AS1-EVs derived from ECA-109 cells upregulated the expression of PD-L1 in macrophages. Data depicts the mean ± SD and are representative of three independent experiments. IR, irradiation, NR, sham IR, CM, conditioned medium, ns no significance, * $p < 0.05$, ** $p < 0.01$, *** $p < 0.001$, ****$p < 0.0001$.

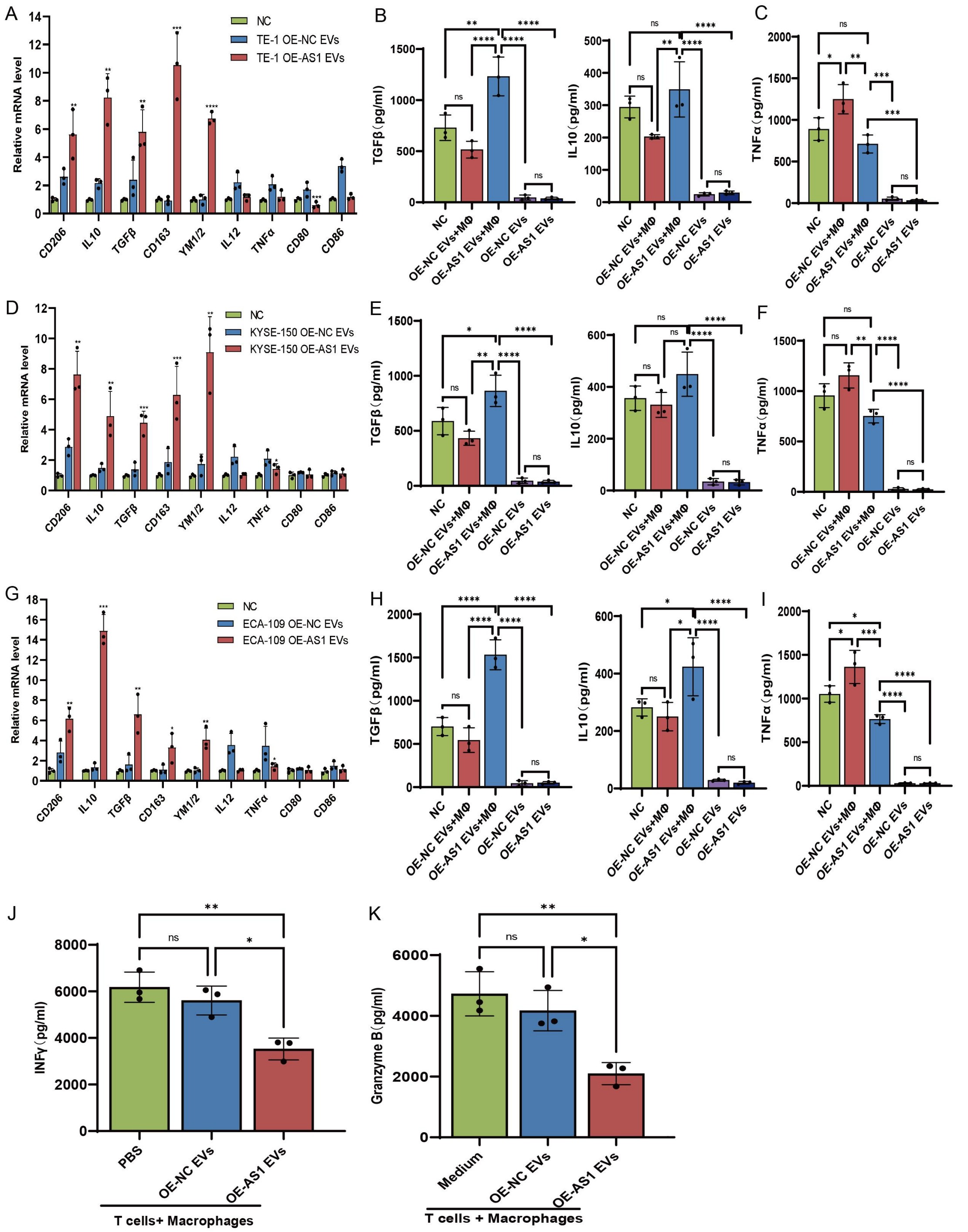

Figure S4 DYNLL1-AS1 enriched in EVs derived from irradiated ESCC cells foster immunosuppressive macrophages formation. A, qPCR analysis was performed to detect the expression of M1 and M2 markers in macrophages treated with NC (PBS), OE-NC EVs, OE-AS1 EVs of TE-1 cells. **B,** ELISA assay of TGFβ and IL10 in macrophages after being co-cultured with NC (PBS), OE-NC EVs, OE-AS1 EVs of TE-1 cells for 24 h. **C,** ELISA assay of TNFα in macrophages

after being co-cultured with NC (PBS), OE-NC EVs, OE-AS1 EVs of TE-1 cells for 24 h. **D,** qPCR analysis was performed to detect the expression of M1 and M2 markers in macrophages treated with NC (PBS), OE-NC EVs, OE-AS1 EVs of KYSE-150 cells. **E,** ELISA of TGFβ and IL10 in macrophages after being co-cultured with NC (PBS), OE-NC EVs, OE-AS1 EVs of KYSE-150 cells for 24 h. **F,** ELISA of TNFα in macrophages after being co-cultured with NC (PBS), OE-NC EVs, OE-AS1 EVs of KYSE-150 cells for 24 h. **G,** qPCR analysis was performed to detect the expression of M1 and M2 markers in macrophages treated with NC (PBS), OE-NC EVs, OE-AS1 EVs of ECA-109 cells. **H,** ELISA of TGFβ and IL10 in macrophages after being co-cultured with NC (PBS), OE-NC EVs, OE-AS1 EVs of ECA-109 cells for 24 h. **I,** ELISA of TNFα in macrophages after being co-cultured with NC (PBS), OE-NC EVs, OE-AS1 EVs of ECA-109 cells for 24 h. **J, K,** ELISA detected T cell production of INF γ (**J**) and Granzyme B (**K**) after cocultured with macrophages pretreated with OE-AS1 EVs, OE-NC-EVs and PBS. Data depicts the mean ± SD and are representative of three independent experiments. ns no significance, * $p < 0.05$, ** $p < 0.01$, *** $p < 0.001$, ****$p < 0.0001$.

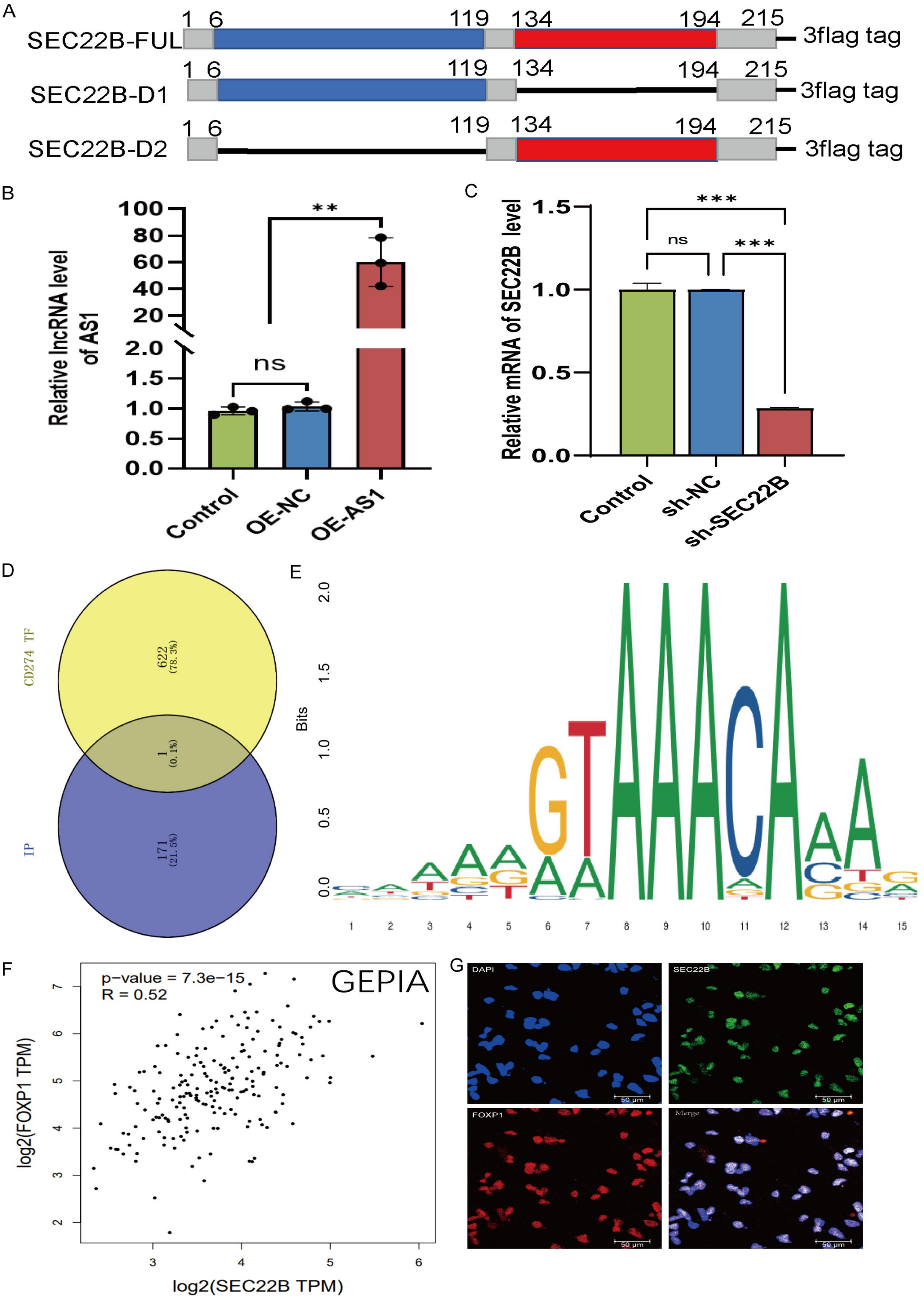

Figure S5 DYNLL1-AS1 targeted SEC22B regulate PD-L1 expression in macrophages via FOXP1. A, Schematic depicting full length and fragmental SEC22B proteins. SEC22B D1: delete 134-194 amino acids; SEC22B D2: delete 6-119 amino acids. **B,** qPCR analysis the expression of

DYNLL1-AS1 in macrophages of OE-NC and OE-DYNLL1-AS1. **C,** qPCR analysis the expression of SEC22B in macrophages of sh-NC and sh-SEC22B. **D,** Venny plot showed the protein interacted with SEC22B using mass spectrum assay and PD-L1 transcription factor predicted using JASPAR database. **E,** The FOXP1 binding site was predicted in the promotor of PD-L1 by JASPAR. **F,** The relationship between SEC22B and FOXP1 based on the expression status according to GEPIA dataset. **G,** Immunofluorescence assays were performed to detect the endogenous co-localization of SEC22B and FOXP1 in macrophages using anti-SEC22B and anti-FOXP1. DAPI-stained nuclei: blue; SEC22B: green; FOXP1: red; merge: superimposed signals of DAPI, SEC22B and FOXP1. Scale bars = 50 μm. Data depict the mean ± SD and are representative of three independent experiments. ns no significance, * $p < 0.05$, ** $p < 0.01$, *** $p < 0.001$, ****$p < 0.0001$.

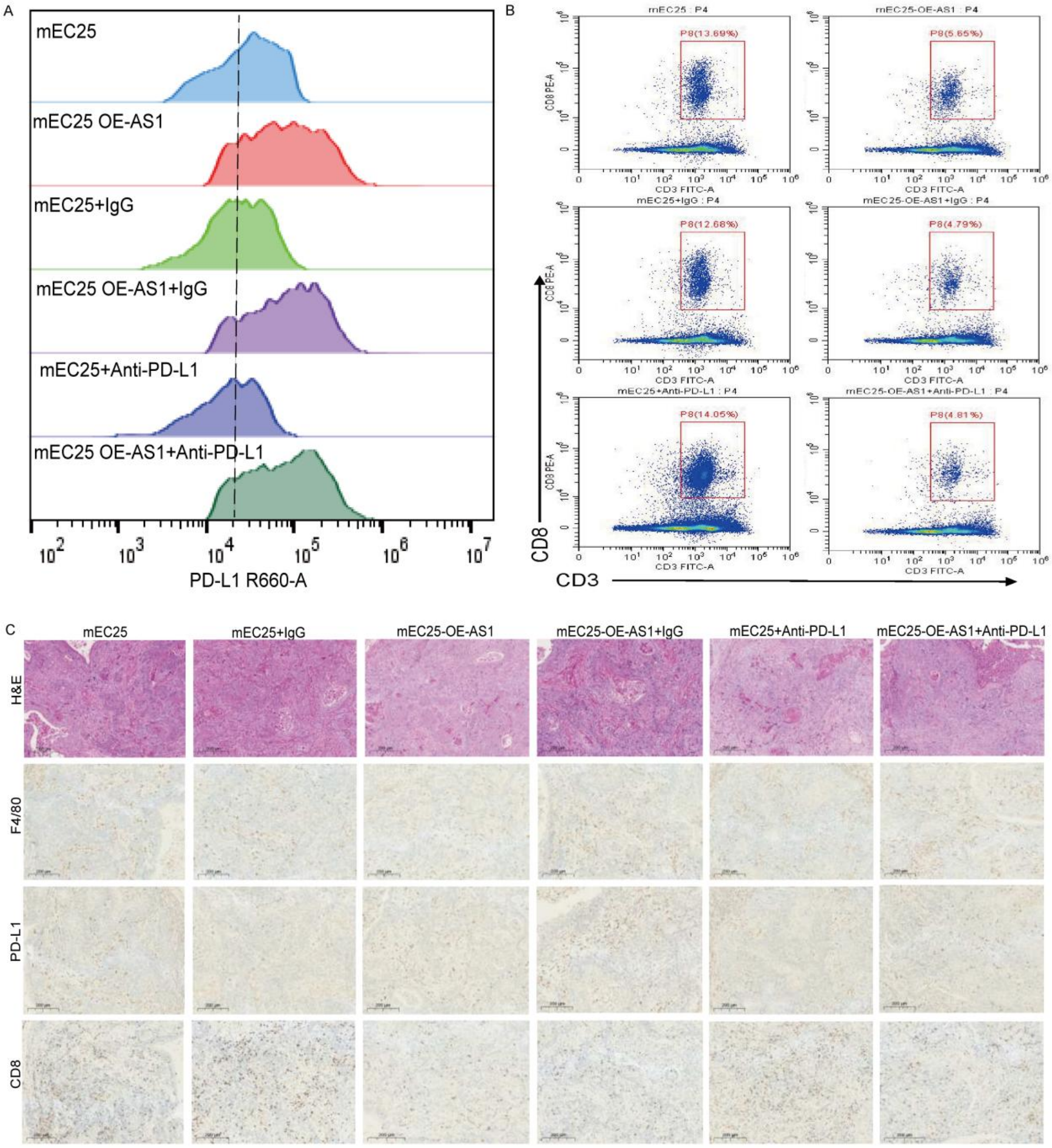

Figure S6 Upregulation of DYNLL1-AS1 in cancer cells inhibit the efficacy of immunotherapy for ESCC in vivo. A, Flow cytometry assay the expression of PD-L1$^+$ TAMs in the tumor tissue. **B,** Flow cytometry assay the expression of CD3$^+$CD8$^+$ T cells in the tumor tissue. **C,** HE and IHC staining were performed to detect the expression levels of F4/80, PD-L1 and CD8 in the implanted tumors.

Supplementary Table 1. The specific primers used for qPCR

Gene	Sequence
ARG1	Foward: 5’-GGTTTTTGTTGTTGCGGTGTTC-3’<br>Reverse: 5’-CTGGGATACTGATGGTGGGATGT-3’
CD163	Forward: 5’-TTTGTCAACTTGAGTCCCTTCAC-3’<br>Reverse: 5’-TCCCGCTACACTTGTTTTCAC-3’
CD68	Forward: 5’-CTTCTCTCATTCCCCTATGGACA-3’<br>Reverse: 5’-GAAGGACACATTGTACTCCACC-3’
TGFβ	Forward: 5’-CAATTCCTGGCGATACCTCAG-3’<br>Reverse: 5’-GCACAACTCCGGTGACATCAA-3’
IL10	Forward: 5’-GACTTTAAGGGTTACCTGGGTTG-3’<br>Reverse: 5’-TCACATGCGCCTTGATGTCTG-3’
IL1β	Forward: 5’-ATGATGGCTTATTACAGTGGCAA-3’<br>Reverse: 5’-GTCGGAGATTCGTAGCTGGA-3’
iNOS	Forward: 5’-TTCAGTATCACAAACCTCAGCCAAG -3’<br>Reverse: 5’-TGGACCTGCAAGTTAAAATCCC-3’
TNFα	Forward: 5’-CCTCTCTCTAATCAGCCCTCTG-3’<br>Reverse: 5’-GAGGACCTGGGAGTAGATGAG-3’
Ym1/2	Forward: 5’-AGGTCACCATTGACAGCAGC-3’<br>Reverse: 5’-ATCCTCCTGACCTCGGAACA-3’
IL12	Forward: 5’-GCGGAGCTGCTACACTCTC -3’<br>Reverse: 5’-CCATGACCTCAATGGGCAGAC-3’
CD80	Forward: 5’-GGCCCGAGTACAAGAACCG -3’<br>Reverse: 5’-TCGTATGTGCCCTCGTCAGAT-3’
CD86	Forward: 5’-CTGCTCATCTATACACGGTTACC -3’<br>Reverse: 5’-GGAAACGTCGTACAGTTCTGTG -3’
GAPDH	Forward: 5'-CTGGGCTACACTGAGCACC-3'<br>Reverse: 5'-AAGTGGTCGTTGAGGGCAATG-3'
β-Actin	Forward: 5'-CATGTACGTTGCTATCCAGGC-3'<br>Reverse: 5'-CTCCTTAATGTCACGCACGAT-3'
CD206	Forward: 5'-GGGTTGCTATCACTCTCTATGC-3'<br>Reverse: 5'-TTTCTTGTCTGTTGCCGTAGTT-3'
DYNLL1-AS1	Forward:5'-TGAGATGATACACATAGAGAAT-3'<br>Reverse:5'-CTACAGGAACACTAATGATAAG-3'
RP11-175K6.1	Forward: 5'-GACTATGCTCCTCTTCTA-3'<br>Reverse: 5'-TGATGTCGTGCTATTGTA-3'
SEC22B	Forward: 5'-AGAAGTTGGCTTTTGCCTACC-3'<br>Reverse: 5'-CACGACTGTCAATGTAGAGCTT-3'
U6	Forward: 5'-ATTGGAACGATACAGAGAAGATT-3'<br>Reverse: 5'-GGAACGCTTCACGAATTTG-3'
PD-L1	Forward: 5'-TGGCATTTGCTGAACGCATTT-3'<br>Reverse: 5'-TGCAGCCAGGTCTAATTGTTTT-3'

Supplementary Table 2. List of antibodies

Antibody	Manufacturer	Catalog	Experimental concentration
Anti-SEC22B antibody	Santa Cruz Biotechnology (Shanghai, China)	sc101267	WB 1:500 Co-IP 2ug IF 1:100
CD206/MRC1(E6T5J)XP Rabit mAb	Cell signaling Technology (TX, USA)	#Q61830	WB 1:1000 IHC 1:400
PD-L1(E1L3N)XP Rabbit mAb	Cell signaling Technology (TX, USA)	Q9NZQ7	WB 1:1000 IHC 1:100
CD68 (E3O7V) Rabbit mAb	Cell signaling Technology (TX, USA)	#97778	IHC 1:100
CD8α (D4W2Z) XP® Rabbit mAb	Cell signaling Technology (TX, USA)	#98941	IHC 1:300
Rabbit monoclonal [EPR26484-79] to FOXP1	Abcam Technology (MA, USA)	ab314488	Co-IP 1:30
YKDDDDK tag Monoclonal antibody (Binds to FLAG® tag epitope)	Proteintech Group (Shanghai, China)	66008-4-ig	RIP 4ug WB 1:5000
Anti-Rabbit IgG	Cell signaling Technology (TX, USA)	#3420S	Co-IP 1:100
Anti-Mouse IgG	Cell signaling Technology (TX, USA)	#3423S	Co-IP 1:100
CD63 Rabbit mAb	Absin (Shanghai, China)	abs159125	WB 1:1000
TSG101 Rabbit mAb	Absin (Shanghai, China)	abs159883	WB 1:1000
FITC anti-human CD14 Antibody	Biolegend (CA, USA)	325604	Flow Cyt: 5 ul/test
APC Mouse Anti-Human CD11b	BD Biosciences (NY, USA)	550019	Flow Cyt: 20 ul/test
BV421 Mouse Anti-Human CD274	BD Biosciences (NY, USA)	563738	Flow Cyt: 5 ul/test
PE Mouse Anti-Human CD206	BD Biosciences (NY, USA)	555954	Flow Cyt: 20 ul/test
FITC anti-mouse CD3ε	Biolegend (CA, USA)	100305	Flow Cyt: 0.25ug/test
APC anti-mouse CD8a	Biolegend (CA, USA)	100713	Flow Cyt: 0.25ug/test
PE anti-mouse F4/80	Biolegend (CA, USA)	123110	Flow Cyt: 1ug/test
FITC anti-mouse CD45 Antibody	Biolegend (CA, USA)	157213	Flow Cyt: 0.25ug/test
PerCP/Cyanine5.5 anti-mouse CD206(MMR) Antibody	Biolegend (CA, USA)	141716	Flow Cyt: 0.4 ug/test
APC anti-mouse CD274	Biolegend (CA, USA)	124311	Flow Cyt: 0.25ug/test

(B7-H1, PD-L1) Antibody			
Donkey anti-Mouse IgG-AlexaFlour 488	Absin (Shanghai, China)	abs20014	IF 1:200
FOXP1 Rabbit mAb	Abclonal (Wuhan, China)	A23442	WB 1:1000 IF 1:100
Anti-Rabbit IgG Cy3	ThermoFisher (MA, USA)	A10520	IF 1:200
Anti-Mouse IgG FITC	ThermoFisher (MA, USA)	F2761	IF 1:200

Supplementary Table 3. The Mass spectrometry identification of proteins pulled down by DYNLL1-AS1

Gene Name	Coverage [%]	# Peptides	# PSMs	# Unique Peptides	# AAs	MW [kDa]	calc. pI
ALDOA	17	7	7	7	364	39.4	8.09
YWHAZ	32	6	6	5	245	27.7	4.79
EEF1G	16	9	9	9	437	50.1	6.67
IFIT3	13	5	6	5	490	56	5.2
HNRNPAB	9	3	3	2	332	36.2	8.21
STT3A	1	1	1	1	705	80.5	8.07
RPL3	3	2	2	2	403	46.1	10.18
EEF1D	7	2	2	2	281	31.1	5.01
RCC1	5	1	1	1	421	44.9	7.52
APEX1	5	1	1	1	318	35.5	8.12
ANXA6	9	4	4	4	673	75.8	5.6
RPL13	5	1	1	1	211	24.2	11.65
ILF3	11	9	10	9	894	95.3	8.76
ME2	6	3	3	3	584	65.4	7.61
FERMT3	3	2	2	2	667	75.9	6.98
XRCC5	6	4	4	4	732	82.7	5.81
RPLP0	16	3	3	3	317	34.3	5.97
HSP90B1	19	15	15	13	803	92.4	4.84
MAP4	2	2	2	2	1152	120.9	5.43
RPL12	15	2	2	2	165	17.8	9.42
ZC3HAV1	3	2	2	2	902	101.4	8.4
EIF2A	2	1	1	1	585	64.9	8.87
HLA-B	6	1	1	1	362	40.4	5.85
HNRNPA3	12	3	4	3	378	39.6	9.01
IFIT1	24	10	10	10	478	55.3	7.2
IGHMBP2	1	1	1	1	993	109.1	8.97
SQOR	5	2	2	2	450	49.9	9.11
ABCF1	2	2	2	2	845	95.9	6.8
AGR2	4	1	1	1	175	20	9
AHSG	2	1	1	1	367	39.3	5.72
DDX3X	8	5	5	5	662	73.2	7.18
RPSA	10	2	2	2	295	32.8	4.87
BUB3	3	1	1	1	328	37.1	6.84
MAGT1	2	1	1	1	335	38	9.63
PDIA4	7	3	4	3	645	72.9	5.07
PGAM1	4	1	1	1	254	28.8	7.18
HMGB3	7	1	1	1	200	23	8.37

GTPBP4	3	2	2	2	634	73.9	9.5
MIF	8	1	1	1	115	12.5	7.88
CDC5L	3	3	3	3	802	92.2	8.18
TRMT1L	1	1	1	1	733	81.7	7.88
HMGB2	17	3	4	2	209	24	7.81
KPNB1	1	1	1	1	876	97.1	4.78
RTRAF	7	2	2	2	244	28.1	6.65
CCT4	15	7	7	6	539	57.9	7.83
SLC25A6	27	8	8	3	298	32.8	9.74
SSBP1	5	1	1	1	148	17.2	9.6
ANP32A	4	1	1	1	249	28.6	4.09
LGALS1	9	1	2	1	135	14.7	5.5
VIM	66	33	43	32	466	53.6	5.12
STAU1	5	3	3	3	577	63.1	9.44
FSCN1	5	2	2	2	493	54.5	7.24
RPL14	5	1	1	1	215	23.4	10.93
HSP90AA1	13	10	10	6	732	84.6	5.02
ALB	63	35	76	35	609	69.3	6.28
DDX41	1	1	1	1	622	69.8	6.84
MARS1	1	1	1	1	900	101.1	6.16
VCP	5	3	3	3	806	89.3	5.26
SLC16A3	3	1	1	1	465	49.4	7.96
WARS1	3	1	1	1	471	53.1	6.23
H2AZ1	20	3	5	1	128	13.5	10.58
KRT2	49	31	41	21	639	65.4	8
G3BP1	4	2	2	2	466	52.1	5.52
ZNF598	2	1	1	1	904	98.6	8.4
FARSB	2	1	1	1	589	66.1	6.84
SRPRB	5	1	1	1	271	29.7	9.04
PC	3	3	3	3	1178	129.6	6.84
HSPB1	26	4	4	4	205	22.8	6.4
HELZ2	1	2	2	2	2649	294.5	7.49
DDOST	5	2	2	2	456	50.8	6.55
RPL10A	6	1	1	1	217	24.8	9.94
PRSS3	4	1	1	1	304	32.5	7.49
HNRNPA0	16	3	3	3	305	30.8	9.29
BLVRB	5	1	1	1	206	22.1	7.65
SEC61A1	4	2	2	2	476	52.2	8.06
KRT14	20	10	13	3	472	51.5	5.16
VARS1	2	2	2	2	1264	140.4	7.59
H1-10	10	2	2	2	213	22.5	10.76
HRNR	2	2	3	2	2850	282.2	10.04
RPS10	14	2	2	2	165	18.9	10.15

RPL6	3	1	1	1	288	32.7	10.58
DLST	3	1	1	1	453	48.7	8.95
G3BP2	2	1	1	1	482	54.1	5.55
ARPC4	10	2	2	2	168	19.7	8.43
CS	4	2	2	2	466	51.7	8.32
UCHL1	4	1	1	1	223	24.8	5.48
SRRM1	3	2	2	2	904	102.3	11.84
KARS1	19	10	10	10	597	68	6.35
AIFM1	2	1	1	1	613	66.9	8.95
RPL35	26	3	3	3	123	14.5	11.05
SRSF7	4	1	1	1	238	27.4	11.82
RPS20	10	1	1	1	119	13.4	9.94
NSUN2	1	1	1	1	767	86.4	6.77
CMAS	16	7	7	7	434	48.3	7.93
CAPRIN1	3	2	2	2	709	78.3	5.25
KRT19	9	4	5	1	400	44.1	5.14
H3-7	20	3	4	3	136	15.4	11.27
MAGEA8	3	1	1	1	318	35.2	4.77
LCP1	13	7	7	7	627	70.2	5.43
PHGDH	2	1	1	1	533	56.6	6.71
TBL2	17	8	8	8	447	49.8	9.44
LBR	1	1	1	1	615	70.7	9.36
HNRNPR	6	4	4	1	633	70.9	8.13
PSMB6	5	1	1	1	239	25.3	4.92
TMEM33	5	1	1	1	247	28	9.7
CEP170	2	3	3	3	1584	175.2	7.11
GLO1	9	1	1	1	184	20.8	5.31
LSM4	5	1	1	1	139	15.3	9.99
RPS13	32	4	4	4	151	17.2	10.54
ARF4	4	1	1	1	180	20.5	7.14
SHMT2	2	1	1	1	504	56	8.53
HAT1	4	1	1	1	419	49.5	5.69
RPL8	4	1	1	1	257	28	11.03
MT-CO2	4	1	1	1	227	25.5	4.82
RPS8	25	5	5	5	208	24.2	10.32
HERC5	1	1	1	1	1024	116.8	7.65
GPNMB	2	1	1	1	572	63.9	6.64
GPI	6	3	3	3	558	63.1	8.32
HSPA6	9	5	7	0	643	71	6.14
KRT10	45	25	42	22	584	58.8	5.21
S100A8	12	1	1	1	93	10.8	7.03
RAB8A	9	2	2	2	207	23.7	9.07
RPL35A	6	1	1	1	110	12.5	11.06

RPS24	20	2	2	2	133	15.4	10.78
ARHGEF2	2	2	2	2	986	111.5	7.27
RPS27A	30	4	4	4	156	18	9.64
KRT1	55	31	49	26	644	66	8.12
RPL4	5	1	1	1	427	47.7	11.06
TCP1	3	2	2	2	556	60.3	6.11
MTDH	23	11	12	11	582	63.8	9.32
RPS18	19	3	3	3	152	17.7	10.99
H1-5	11	3	3	3	226	22.6	10.92
POR	1	1	1	1	677	76.6	5.58
TUFM	3	1	1	1	452	49.5	7.61
GLUD1	2	1	1	1	558	61.4	7.8
PLCG1	2	1	1	1	1290	148.4	6.05
KRT6A	14	11	12	2	564	60	8
TMED10	4	1	1	1	219	25	7.44
KPRP	2	1	1	1	579	64.1	8.27
LRRC59	16	5	5	5	307	34.9	9.57
AIMP1	7	1	2	1	312	34.3	8.43
SCAMP3	5	1	1	1	347	38.3	7.64
SPATS2L	2	1	1	1	558	61.7	9.64
SRP9	13	1	1	1	86	10.1	7.97
RPS6	28	9	11	9	249	28.7	10.84
DARS2	1	1	1	1	645	73.5	8.02
SLAIN2	3	1	1	1	581	62.5	9.45
PRDX6	4	1	1	1	224	25	6.38
PLEC	19	81	85	81	4684	531.5	5.96
ANP32B	3	1	1	1	251	28.8	4.06
CES1	4	2	2	2	567	62.5	6.6
MX2	9	5	6	5	715	82	8.76
MNDA	37	16	25	16	407	45.8	9.76
CYFIP2	1	1	1	1	1278	148.3	7.31
PPP1CA	3	1	1	1	330	37.5	6.33
B2M	8	1	1	1	119	13.7	6.52
RPL23	11	2	2	2	140	14.9	10.51
LARS1	1	1	1	1	1176	134.4	7.3
RPN2	10	4	4	4	631	69.2	5.69
DRG1	3	1	1	1	367	40.5	8.9
P4HB	20	9	12	9	508	57.1	4.87
SERPINB2	21	6	7	6	415	46.6	5.63
PHB2	6	2	2	2	299	33.3	9.83
VDAC2	7	1	1	1	294	31.5	7.56
SERBP1	10	3	3	3	408	44.9	8.65
SLC35E1	6	1	1	1	410	44.7	9.79

MDH2	24	6	6	6	338	35.5	8.68
SLC25A5	27	8	8	3	298	32.8	9.69
HSP90AB1	16	11	11	5	724	83.2	5.03
TKT	12	5	5	5	623	67.8	7.66
PRPF19	6	3	3	3	504	55.1	6.61
SERPINA1	8	3	4	3	418	46.7	5.59
FLG2	0	1	1	1	2391	247.9	8.31
PRRC2C	0	1	1	1	2896	316.7	9.13
HNRNPUL1	1	1	1	1	856	95.7	6.92
NACA	1	1	1	1	2078	205.3	9.58
DSG1	2	1	1	1	1049	113.7	5.03
RPL19	5	1	1	1	196	23.5	11.47
ARPC5	8	1	1	1	151	16.3	5.67
CIRBP	13	2	2	2	172	18.6	9.51
KRT85	1	1	1	1	507	55.8	6.55
ZC3H15	5	1	1	1	426	48.6	5.31
RPS11	38	7	9	7	158	18.4	10.3
SERPINC1	2	1	1	1	464	52.6	6.71
CFL1	22	3	3	3	166	18.5	8.09
CAPG	2	1	1	1	348	38.5	6.19
HSPE1	28	3	3	3	102	10.9	8.92
RPS27	10	1	1	1	84	9.5	9.45
MSN	10	6	7	6	577	67.8	6.4
UPF1	1	1	1	1	1129	124.3	6.61
RPL13A	11	2	2	2	203	23.6	10.93
PGK1	14	4	4	4	417	44.6	8.1
HSD17B4	8	3	3	3	736	79.6	8.84
LRPPRC	1	1	1	1	1394	157.8	6.13
VDAC1	18	4	4	4	283	30.8	8.54
HNRNPH1	10	3	3	3	449	49.2	6.3
CCT8	6	3	3	3	548	59.6	5.6
RPL31	26	3	3	3	125	14.5	10.54
CLIC1	8	2	2	2	241	26.9	5.17
QARS1	11	8	8	8	775	87.7	7.15
PCBP2	10	3	3	2	365	38.6	6.79
LRPAP1	3	1	1	1	357	41.4	8.78
HSPA8	28	17	19	12	646	70.9	5.52
ASPH	2	1	1	1	758	85.8	5.01
GSN	2	1	1	1	782	85.6	6.28
SEC22B	5	1	1	1	215	24.7	8.51
A2M	1	2	2	2	1474	163.2	6.46
IFIT2	2	1	1	1	472	54.6	6.76
RBMXL3	1	1	1	1	1067	114.9	9.1

ACACA	0	1	1	1	2346	265.4	6.37
SYNCRIP	25	13	13	10	623	69.6	8.59
EEF1A1	27	9	15	9	462	50.1	9.01
GFPT2	1	1	1	1	682	76.9	7.37
KRT84	3	3	3	1	600	64.8	7.56
ACTB	50	14	27	7	375	41.7	5.48
HNRNPU	14	10	11	10	825	90.5	6
LDHB	19	5	5	5	334	36.6	6.05
MCCC2	5	2	2	2	563	61.3	7.68
SRSF8	3	1	1	1	282	32.3	11.72
SUB1	9	1	1	1	127	14.4	9.6
DDX1	2	2	2	2	740	82.4	7.23
PDS5B	1	2	2	2	1447	164.6	8.47
RPS7	27	6	6	6	194	22.1	10.1
CKAP5	3	5	5	5	2032	225.4	7.8
SSRP1	1	1	1	1	709	81	6.87
SETSIP	3	1	1	1	302	34.9	4.31
PHB1	16	4	4	4	272	29.8	5.76
GLG1	1	1	1	1	1179	134.5	6.9
RPS3	12	3	3	3	243	26.7	9.66
FUS	3	1	1	1	526	53.4	9.36
CCDC124	4	1	1	1	223	25.8	9.54
ANXA2	40	12	13	12	339	38.6	7.75
HADHA	1	1	1	1	763	82.9	9.04
PCBP1	9	3	3	2	356	37.5	7.09
LMNA	24	15	15	15	664	74.1	7.02
YBX1	18	4	4	4	324	35.9	9.88
KHSRP	2	1	1	1	711	73.1	7.3
GAPDH	32	9	13	9	335	36	8.46
IGF2BP3	3	1	1	1	579	63.7	8.87
YWHAG	17	4	4	2	247	28.3	4.89
EIF5B	9	7	7	7	1220	138.7	5.49
ILF2	26	9	13	9	390	43	5.26
HSPA5	36	21	24	19	654	72.3	5.16
DAD1	28	3	3	3	113	12.5	7.08
JUP	1	1	1	1	745	81.7	6.14
RPL11	12	2	2	2	178	20.2	9.6
RPS23	23	3	3	3	143	15.8	10.49
MCUB	8	1	1	1	336	39.1	9.16
EIF2S3	33	10	12	10	472	51.1	8.4
RPL38	36	2	2	2	70	8.2	10.1
HNRNPA2B1	20	7	8	5	353	37.4	8.95
COL1A1	2	1	1	1	1464	138.8	5.8

ECH1	5	2	2	2	328	35.8	8
EPRS1	11	13	13	13	1512	170.5	7.33
MAPRE1	3	1	1	1	268	30	5.14
RPL27	7	1	1	1	136	15.8	10.56
ACADVL	1	1	1	1	655	70.3	8.75
MOGS	2	1	1	1	837	91.9	8.9
RPL7A	3	1	1	1	266	30	10.61
RCC2	3	1	1	1	522	56	8.78
SF1	2	1	1	1	639	68.3	8.98
CACYBP	3	1	1	1	228	26.2	8.25
PFN1	31	3	3	3	140	15	8.27
EIF2S1	20	6	6	6	315	36.1	5.08
PDIA3	14	7	7	7	505	56.7	6.35
H4C16	51	6	8	6	103	11.4	11.36
AIMP2	12	2	3	2	320	35.3	8.22
HNRNPK	21	7	11	7	463	50.9	5.54
EEF1B2	4	1	1	1	225	24.7	4.67
FAU	8	2	2	2	133	14.4	10.17
SRP14	16	2	2	2	136	14.6	10.04
KHDRBS1	5	2	2	2	443	48.2	8.66
RARS1	10	5	5	5	660	75.3	6.68
RPL30	23	2	2	2	115	12.8	9.63
SNRPB	3	1	1	1	240	24.6	11.19
RPL17	22	3	4	3	184	21.4	10.17
NARS1	2	1	1	1	548	62.9	6.25
SFPQ	14	9	9	8	707	76.1	9.44
CAP1	4	1	1	1	475	51.9	8.06
IGLC2	9	1	1	1	106	11.3	7.24
SRSF6	5	2	2	2	344	39.6	11.43
ACAT1	2	1	1	1	427	45.2	8.85
DDX17	12	7	7	4	729	80.2	8.27
LYZ	6	1	1	1	148	16.5	9.16
TRMT6	2	1	1	1	497	55.8	7.55
XRCC6	7	3	3	3	609	69.8	6.64
PPIA	25	4	4	4	165	18	7.81
RPL22	20	3	3	3	128	14.8	9.19
BCAP31	4	1	1	1	246	28	8.44
ATP5F1A	22	11	11	11	553	59.7	9.13
ATP5F1B	13	5	6	5	529	56.5	5.4
CANX	5	3	3	3	592	67.5	4.6
RPN1	12	5	6	5	607	68.5	6.38
PURB	2	1	1	1	312	33.2	5.43
HNRNPL	2	1	1	1	589	64.1	8.22

HTT	0	1	1	1	3142	347.4	6.2
ERO1A	3	1	1	1	468	54.4	5.68
PTDSS1	2	1	2	1	473	55.5	8.43
LDHA	5	2	2	2	332	36.7	8.27
CD44	3	2	2	2	742	81.5	5.33
HSPA1A	10	6	8	2	641	70	5.66
ALYREF	4	1	1	1	257	26.9	11.15
ACTA2	22	8	14	1	377	42	5.39
HARS1	2	1	1	1	509	57.4	5.88
TPI1	35	6	6	6	249	26.7	6.9
HSD17B10	6	1	1	1	261	26.9	7.78
MYH9	4	6	6	6	1960	226.4	5.6
CCT2	9	3	3	3	535	57.5	6.46
CALM1	20	2	2	2	149	16.8	4.22
ATXN2L	1	1	1	1	1075	113.3	8.59
H1-2	25	7	11	7	213	21.4	10.93
RPS4X	35	11	15	11	263	29.6	10.15
TF	6	3	3	3	698	77	7.12
RPS14	15	2	2	2	151	16.3	10.05
U2AF2	4	2	2	2	475	53.5	9.09
CCT6A	3	2	2	2	531	58	6.68
RBM39	5	2	2	2	530	59.3	10.1
RPS25	14	2	2	2	125	13.7	10.11
MBNL1	5	2	2	2	388	41.8	8.9
RPL24	24	4	5	4	157	17.8	11.25
LYAR	7	2	2	2	379	43.6	9.57
EIF3B	1	1	1	1	814	92.4	5
S100A6	9	1	1	1	90	10.2	5.48
ACTN4	13	9	9	9	911	104.8	5.44
PTBP1	18	8	10	8	557	59.6	9.16
HMGB1	18	4	6	3	215	24.9	5.74
NCL	29	23	28	23	710	76.6	4.7
MCCC1	3	2	2	2	725	80.4	7.78
RPS26	13	1	1	1	115	13	11
EEF2	4	3	3	3	858	95.3	6.83
RPS2	18	5	5	5	293	31.3	10.24
MYL6	6	1	1	1	151	16.9	4.65
RRBP1	38	48	57	48	1410	152.4	8.6
PLSCR1	4	1	1	1	318	35	4.94
PA2G4	3	1	1	1	394	43.8	6.55
RPS3A	12	3	3	3	264	29.9	9.73
RPL26	16	3	3	3	145	17.2	10.55
HNRNPD	18	5	6	4	355	38.4	7.81

H2BC26	33	4	6	4	126	13.9	10.32
PRDX1	27	6	6	6	199	22.1	8.13
HADHB	4	2	2	2	474	51.3	9.41
RPS9	41	12	13	12	194	22.6	10.65
SRSF1	4	1	1	1	248	27.7	10.36
HNRNPDL	4	2	2	1	420	46.4	9.57
ADAR	1	2	2	2	1226	136	8.65
TUBA1B	22	8	8	8	451	50.1	5.06
NAMPT	2	1	1	1	491	55.5	7.15
ANXA1	16	4	4	4	346	38.7	7.02
DARS1	19	8	8	8	501	57.1	6.55
NPM1	10	3	4	3	294	32.6	4.78
CLTC	3	3	3	3	1675	191.5	5.69
IARS1	2	2	2	2	1262	144.4	6.15
APOA1	15	4	4	4	267	30.8	5.76
DCD	13	1	1	1	110	11.3	6.54
FAM120A	1	1	1	1	1118	121.8	8.88
SRSF3	18	2	2	2	164	19.3	11.65
RPL15	7	1	1	1	204	24.1	11.62
DHX15	1	1	1	1	795	90.9	7.46
EIF4A2	2	1	1	1	407	46.4	5.48
TMPO	2	1	1	1	694	75.4	7.66
RPL7	4	1	1	1	248	29.2	10.65
DDX5	17	10	10	7	614	69.1	8.92
PABPN1	4	1	1	1	306	32.7	5.06
ISG15	13	2	3	2	165	17.9	7.44
HSPA9	6	3	3	3	679	73.6	6.16
ENO1	33	12	14	12	434	47.1	7.39
BLM	1	1	1	1	1417	158.9	7.49
HSPD1	19	8	10	8	573	61	5.87
ANPEP	1	1	1	1	967	109.5	5.48
PGAM5	8	2	2	2	289	32	8.68
RPL23A	22	4	4	4	156	17.7	10.45
HNRNPM	20	13	15	13	730	77.5	8.7
PDIA6	9	3	3	3	440	48.1	5.08
RPS12	6	1	1	1	132	14.5	7.21
PRKDC	1	5	5	5	4128	468.8	7.12
TXN	12	1	1	1	105	11.7	4.92
ACTR3	5	1	1	1	418	47.3	5.88
COPB2	1	1	1	1	906	102.4	5.27
GOT2	3	1	1	1	430	47.5	9.01
PPIB	21	4	4	4	216	23.7	9.41
TREX1	4	1	1	1	314	33.2	7.93

RPL36A	25	3	3	3	106	12.4	10.58
EFHD2	5	1	1	1	240	26.7	5.2
TUBB	19	6	7	6	444	49.6	4.89
PKM	30	11	13	11	531	57.9	7.84
RBBP4	3	1	1	1	425	47.6	4.89
OAS3	7	8	9	8	1087	121.1	8.4
RAN	5	1	1	1	216	24.4	7.49
IGHG3	3	1	1	1	377	41.3	7.9
APOH	3	1	1	1	345	38.3	7.97
ACO2	1	1	1	1	780	85.4	7.61
DDX39B	7	3	3	3	428	49	5.67
DEFA1B	10	1	1	1	94	10.2	6.99
FMR1	3	2	2	2	632	71.1	7.42
EIF2S2	19	5	6	5	333	38.4	5.8
CCT3	3	2	2	2	545	60.5	6.49
OSTC	8	1	1	1	149	16.8	9.13
PABPC1	11	5	5	5	636	70.6	9.5
NME2	27	4	4	4	152	17.3	8.41
DHX9	12	15	15	15	1270	140.9	6.84
ANXA5	9	3	3	3	320	35.9	5.05
PGD	2	1	1	1	483	53.1	7.23
KRT8	12	7	8	2	483	53.7	5.59
RPL10	6	1	1	1	214	24.6	10.08
NONO	14	7	8	6	471	54.2	8.95
TECR	3	1	1	1	308	36	9.45
RPS15A	32	4	4	4	130	14.8	10.13
CPLANE1	1	1	3	1	3197	361.5	6.99
EIF2AK2	11	6	6	6	551	62.1	8.4
SSB	10	3	3	3	408	46.8	7.12
SUPT16H	1	1	1	1	1047	119.8	5.66
RPS17	32	4	4	4	135	15.5	9.85
CCT5	3	2	2	1	541	59.6	5.66
FABP5	7	1	1	1	135	15.2	7.01
RACK1	3	1	1	1	317	35.1	7.69
MAP1S	5	4	4	4	1059	112.1	7.3
H2AC17	35	5	8	3	130	14.1	10.9
RTN4	1	1	1	1	1192	129.9	4.5
SND1	28	20	23	20	910	101.9	7.17
TRMT61A	3	1	1	1	289	31.4	7.36
SRPK1	2	1	1	1	655	74.3	6.16
KRT9	53	23	34	23	623	62	5.24
KRT16	19	10	13	3	473	51.2	5.05
PTMA	13	1	1	1	111	12.2	3.78

YWHAQ	13	3	3	1	245	27.7	4.78
DSC1	2	1	1	1	894	99.9	5.43
IFI16	14	10	11	10	785	88.2	9.28
CTSD	2	1	1	1	412	44.5	6.54
ATP5ME	14	1	1	1	69	7.9	9.35
TRIM25	7	4	4	4	630	70.9	8.09
CAT	2	1	1	1	527	59.7	7.39
CKAP4	20	10	10	10	602	66	5.92
HNRNPA1	28	9	12	7	372	38.7	9.13
LONP1	1	1	1	1	959	106.4	6.39
FBL	8	2	2	2	321	33.8	10.18
RPS16	7	1	1	1	146	16.4	10.21
PARK7	8	1	1	1	189	19.9	6.79
ARPC2	3	1	1	1	300	34.3	7.36
CALR	10	5	5	5	417	48.1	4.44
SEC11A	4	1	1	1	179	20.6	9.48
CENPF	0	1	1	1	3114	357.3	5.1
ELAVL1	3	1	1	1	326	36.1	9.17
IQGAP1	7	8	8	8	1657	189.1	6.48
KTN1	1	1	1	1	1357	156.2	5.64
KRT5	19	15	16	7	590	62.3	7.74
FAM98A	2	1	1	1	518	55.2	8.95
RPL27A	16	2	2	2	148	16.6	11
EIF4H	5	1	1	1	248	27.4	7.23
IMMT	1	1	1	1	758	83.6	6.48
TNIK	1	1	1	1	1360	154.8	7.17
CYCS	10	1	1	1	105	11.7	9.57
OSBPL8	1	1	1	1	889	101.1	6.96
TOP1	22	15	17	15	765	90.7	9.31
DNAJB1	3	1	1	1	340	38	8.63

Abbreviation: PSMs, Peptide spectrum matches; AAs, Animo acids; MW, Molecular weight; calc.pI, calculated isoelectric point.

Supplementary Table 4. The Mass spectrometry identification of proteins pulled down by SEC22B

Gene Name	Coverage [%]	Peptides	PSMs	Unique Peptides	AAs	MW [kDa]	calc. pI	Score Mascot: Mascot
ALDOA	6	3	3	3	364	39.4	8.09	84
YWHAZ	8	2	2	2	245	27.7	4.79	38
EEF1G	2	1	1	1	437	50.1	6.67	26
RPL3	3	2	2	2	403	46.1	10.18	64
ANXA6	2	1	1	1	673	75.8	5.6	39
RPL13	5	1	1	1	211	24.2	11.65	67
RAB1A	4	1	1	1	205	22.7	6.21	22
RPLP0	7	1	1	1	317	34.3	5.97	33
HSP90B1	9	6	6	5	803	92.4	4.84	254
RPL12	5	1	1	1	165	17.8	9.42	49
EWSR1	1	1	1	1	656	68.4	9.33	0
TGM3	1	1	1	1	693	76.6	5.86	28
DDX3X	2	1	1	1	662	73.2	7.18	56
TRIM56	3	1	1	1	755	81.4	7.74	0
ERLIN1	9	3	5	1	348	39.1	7.87	116
LGALS1	23	3	3	3	135	14.7	5.5	38
VIM	8	4	4	3	466	53.6	5.12	136
HSP90AA1	11	8	9	1	732	84.6	5.02	317
ALB	7	5	19	5	609	69.3	6.28	527
VCP	2	1	1	1	806	89.3	5.26	41
CCAR2	1	1	1	1	923	102.8	5.22	63
KRT2	17	10	14	7	639	65.4	8	517
CHI3L1	2	1	1	1	383	42.6	8.46	48
KRT14	16	8	13	3	472	51.5	5.16	363
HRNR	1	1	1	1	2850	282.2	10.04	29
TMSB4X	59	2	4	2	44	5.1	5.06	134
COL3A1	1	1	1	1	1466	138.5	6.61	49
AP5Z1	1	1	1	1	807	88.5	7.01	27
RPS20	9	1	1	1	119	13.4	9.94	46
ERP29	4	1	1	1	261	29	7.31	49
KRT19	9	5	8	1	400	44.1	5.14	167
SIX5	1	1	1	1	739	74.5	4.96	48
ALDOC	6	1	2	1	364	39.4	6.87	44
LCP1	3	2	2	2	627	70.2	5.43	62
HYOU1	1	1	1	1	999	111.3	5.22	84
TUFM	3	1	1	1	455	49.8	7.61	35
RPS8	6	1	1	1	208	24.2	10.32	42

MCM3	1	1	1	1	808	90.9	5.77	0
FOXP1	2	1	1	1	677	75.3	6.67	57
KRT10	21	14	26	10	584	58.8	5.21	926
RPS24	6	1	1	1	133	15.4	10.78	34
RPS27A	24	3	3	3	156	18	9.64	50
KRT1	31	20	54	16	644	66	8.12	1627
RPS18	24	4	8	4	152	17.7	10.99	202
H1-5	21	5	5	4	226	22.6	10.92	136
KRT6B	13	7	11	2	564	60	8	330
RPS6	8	2	2	2	249	28.7	10.84	104
PRDX6	5	1	1	1	224	25	6.38	58
RPSA2	4	1	1	1	295	32.9	4.87	20
MYL12B	17	2	2	2	172	19.8	4.84	68
P4HB	22	11	13	11	508	57.1	4.87	399
MDH2	13	3	3	3	338	35.5	8.68	90
HSP90AB1	16	11	12	3	724	83.2	5.03	403
TKT	1	1	1	1	623	67.8	7.66	42
PABPC4	2	1	1	1	644	70.7	9.26	35
H2BC1	28	4	7	4	127	14.2	10.32	194
HBZ	5	1	1	1	142	15.6	8.21	0
TRIM28	2	2	2	2	835	88.5	5.77	69
RPL19	5	1	1	1	196	23.5	11.47	54
RPS11	5	1	1	1	158	18.4	10.3	41
CFL1	19	2	2	2	166	18.5	8.09	79
CAPG	3	1	1	1	348	38.5	6.19	51
HSPE1	18	2	2	2	102	10.9	8.92	71
MSN	12	7	8	6	577	67.8	6.4	175
SLK	1	1	4	1	1235	142.6	5.15	68
CCT8	3	1	1	1	548	59.6	5.6	57
CLIC1	7	1	1	1	241	26.9	5.17	68
EZR	3	2	2	1	586	69.4	6.27	77
HSPA8	15	7	9	6	646	70.9	5.52	291
SEC22B	41	8	26	8	215	24.7	8.51	947
GRN	21	11	17	11	593	63.5	6.83	302
RPL18	6	1	1	1	188	21.6	11.72	32
EEF1A1	11	4	6	4	462	50.1	9.01	176
ACTB	47	13	47	6	375	41.7	5.48	1502
HNRNPU	3	3	3	3	825	90.5	6	64
SUB1	7	1	1	1	127	14.4	9.6	23
RAB7A	3	1	1	1	207	23.5	6.7	44
SETSIP	3	1	1	1	302	34.9	4.31	35
RPS3	4	1	1	1	243	26.7	9.66	43
HADHA	1	1	1	1	763	82.9	9.04	43

LMNA	4	2	2	2	664	74.1	7.02	53
GAPDH	31	8	10	8	335	36	8.46	259
HSPA5	18	10	12	9	654	72.3	5.16	331
RAC2	7	1	1	1	192	21.4	7.61	26
RPL11	8	1	1	1	178	20.2	9.6	29
HNRNPA2B1	3	1	1	1	353	37.4	8.95	36
COL1A1	1	2	2	2	1464	138.8	5.8	67
ECH1	3	1	1	1	328	35.8	8	51
ERLIN2	14	4	6	2	339	37.8	5.62	164
PFN1	19	2	2	2	140	15	8.27	73
PDIA3	9	4	4	4	505	56.7	6.35	150
H4C1	39	4	7	4	103	11.4	11.36	187
HNRNPK	3	2	2	2	463	50.9	5.54	61
WDR11	1	2	2	2	1224	136.6	6.92	41
TRIP11	1	2	2	2	1979	227.4	5.26	57
SLC25A31	3	1	1	1	315	35	9.89	40
SFPQ	1	1	1	1	707	76.1	9.44	43
CAP1	29	12	16	12	475	51.9	8.06	371
RPL32	5	1	1	1	135	15.9	11.33	35
PPIA	32	5	5	5	165	18	7.81	179
GTF2I	2	2	2	2	998	112.3	6.39	69
ATP5F1A	14	7	7	7	553	59.7	9.13	180
ATP5F1B	7	3	3	3	529	56.5	5.4	35
H3C1	5	1	1	1	136	15.4	11.12	48
ACTA2	22	8	34	1	377	42	5.39	902
TPI1	6	1	1	1	249	26.7	6.9	54
H1-4	8	2	2	1	219	21.9	11.03	88
MYH9	17	23	23	23	1960	226.4	5.6	1034
CALM1	9	1	1	1	149	16.8	4.22	24
RPL5	5	1	1	1	297	34.3	9.72	37
RPS4X	3	1	1	1	263	29.6	10.15	37
RPS25	8	1	1	1	125	13.7	10.11	19
ITGB2	3	2	2	2	769	84.7	6.95	77
NCL	9	7	8	7	710	76.6	4.7	166
EEF2	3	3	3	3	858	95.3	6.83	86
MYL6	26	3	3	3	151	16.9	4.65	126
H2AX	6	1	1	1	143	15.1	10.74	41
RPS3A	3	1	1	1	264	29.9	9.73	46
RPL26	6	1	1	1	145	17.2	10.55	33
HNRNPD	5	2	2	2	355	38.4	7.81	39
HLA-A	2	1	1	1	365	40.8	6	55
RPS5	7	1	1	1	204	22.9	9.72	82
TMSB10	32	1	1	1	44	5	5.36	50

TUBA1B	15	5	5	5	451	50.1	5.06	169
NPM1	9	3	4	3	294	32.6	4.78	95
CLTC	1	2	2	2	1675	191.5	5.69	80
CORO1A	11	5	5	5	461	51	6.68	101
S100A11	9	1	1	1	105	11.7	7.12	52
FCGR1A	11	5	6	5	374	42.6	7.97	180
GSTP1	9	2	2	2	210	23.3	5.64	63
ARPC1B	2	1	1	1	372	40.9	8.35	39
HLA-C	2	1	1	1	366	40.6	6.04	65
FH	1	1	1	1	510	54.6	8.76	37
HSPA9	11	6	6	6	679	73.6	6.16	201
ENO1	18	7	8	7	434	47.1	7.39	252
HSPD1	11	5	6	5	573	61	5.87	186
RPL23A	5	1	1	1	156	17.7	10.45	44
HNRNPM	3	2	2	2	730	77.5	8.7	78
PDIA6	2	1	1	1	440	48.1	5.08	44
PSMB8	3	1	1	1	276	30.3	7.43	41
ACTR3	2	1	1	1	418	47.3	5.88	33
MYO1D	1	1	1	1	1006	116.1	9.39	44
MDH1	5	2	2	2	334	36.4	7.36	40
PPIB	3	1	1	1	216	23.7	9.41	32
TUBB	16	5	5	2	444	49.6	4.89	178
PKM	8	3	3	3	531	57.9	7.84	47
IMPDH2	2	1	1	1	514	55.8	6.9	44
VASP	2	1	1	1	380	39.8	8.94	54
RAN	15	3	4	3	216	24.4	7.49	66
TUBB4B	15	5	5	2	445	49.8	4.89	178
SERPINH1	6	1	1	1	418	46.4	8.69	24
GDI2	2	1	1	1	445	50.6	6.47	33
KRT8	7	4	5	1	483	53.7	5.59	99
CAPZA1	3	1	1	1	286	32.9	5.69	63
FOXQ1	12	1	1	1	403	41.5	9.44	0
RACK1	2	1	1	1	317	35.1	7.69	47
H2AC11	20	2	2	2	130	14.1	10.9	101
CA2	15	3	4	3	260	29.2	7.4	118
PPT1	3	1	1	1	306	34.2	6.52	26
KRT9	15	10	16	9	623	62	5.24	514
EDRF1	1	1	1	1	1238	138.4	6.33	0
PTMA	25	2	2	2	111	12.2	3.78	93
SH3BGRL3	11	1	1	1	93	10.4	4.93	77
TAPBP	2	1	1	1	448	47.5	6.99	15
TOPAZ1	1	1	1	1	1692	190.8	7.87	0
RPS16	13	2	2	2	146	16.4	10.21	90

LGALS9B	2	1	1	1	356	39.6	9.52	58
CALR	5	2	2	2	417	48.1	4.44	60
KRT5	7	4	4	1	590	62.3	7.74	136
EIF4H	3	1	1	1	248	27.4	7.23	27
PRSS3P2	12	1	2	1	247	26.5	6.01	141

Abbreviation: PSMs, Peptide spectrum matches; AAs, Animo acids; MW, Molecular weight.

Supplementary Table 5. Clinicopathological factors of ESCC patients with neoadjuvant immunotherapy

Clinicopathological factors	Number of patients	%
Age(years)[range 47-74; median 67]		
≤60	7	30.4
>60	16	69.6
Gender		
Male	19	82.6
Female	4	17.4
Tumor size (cm)		
≤3	14	60.9
>3	9	39.1
Tumor location		
Upper	1	4.3
Middle	12	52.2
Lower	10	43.5
Tumor differentiation		
Well	2	8.7
Modest	14	60.9
Poor	7	30.4
pN		
N0	14	60.9
N1	5	21.7
N2	3	13.0
N3	1	4.3
pT		
T1	7	30.4
T2	8	34.8
T3	8	34.8
T4	0	0
pTNM stage		
I	5	21.7
II	11	47.8
III	6	26.1
IV	1	4.3
Immunotherapy		
Pembrolizumab	21	91.3
Others	2	8.7
Tumor regression grade, TRG		
1	4	17.4
2	10	43.5
3	9	39.1

Supplementary Table 6. Association between clinical factors and DYNLLA-AS1 expression in ESCC patients with neoadjuvant immunotherapy

Clinicopathological factors	N	DYNLL1-AS1 Expression		*p* value
		Low	High	
Age (years)				0.405
≤60	7	5	2	
>60	16	8	8	
Gender				1.000
Male	19	11	8	
Female	4	2	2	
Tumor size (cm)				0.029
≤3	14	5	9	
>3	9	8	1	
Tumor location				0.505
Upper	1	0	1	
Middle	12	7	5	
Lower	10	6	4	
Tumor differentiation				0.634
Well	2	1	1	
Modest	14	9	5	
Poor	7	3	4	
pN				0.281
N0+N1	19	12	7	
N2+N3	4	1	3	
pT				0.580
T1+T2	13	8	5	
T3+T4	10	5	5	
pTNM stages				0.417
I +II	14	9	5	
III+IV	9	4	5	
Tumor regression grade, TRG				0.017
1	4	1	3	
2	10	9	1	
3	9	3	6	

Supplementary Table 7. Clinicopathological factors of patients with ESCC undergoing radical esophagectomy

Clinicopathological factors	Number of patients	%
Age(years)[range 39-74; median 60]		
≤60	43	57.3
>60	32	42.7
Gender		
Male	62	82.7
Female	13	17.3
Tumor size (cm)		
≤3	38	50.3
>3	37	49.7
Tumor location		
Upper	3	4.0
Middle	43	57.3
Lower	29	38.7
Tumor differentiation		
Well	7	9.3
Modest	41	54.7
Poor	27	36.0
pN		
N0	30	40.0
N1	27	36.0
N2	11	14.7
N3	7	9.3
pT		
T1	18	24.0
T2	15	20.0
T3	27	36.0
T4	15	20.0
pTNM stage		
I	11	14.7
II	24	32.0
III	24	32.0
IV	16	21.3
Adjuvant therapy		
Yes	30	40.0
No	45	60.0

Supplementary Table 8. Association between clinical factors and DYNLLA-AS1 expression in patients with ESCC undergoing radical esophagectomy

Clinicopathological factors	N	DYNLL1-AS1 Expression		*p* value
		Low	High	
Age (years)				0.400
≤60	43	27	16	
>60	32	16	16	
Gender				0.780
Male	62	36	26	
Female	13	7	6	
Tumor size (cm)				0.049
≤3	38	26	12	
>3	37	17	20	
Tumor location				0.917
Upper	3	2	1	
Middle	43	25	18	
Lower	29	16	13	
Tumor differentiation				0.497
Well	14	10	4	
Modest	37	20	17	
Poor	24	13	11	
pN				0.018
N0+N1	57	37	20	
N2+N3	18	6	12	
pT				0.017
T1+T2	33	24	9	
T3+T4	42	19	23	
pTNM stages				0.005
I +II	35	26	9	
III+IV	40	17	23	

Supplementary Table 9. Clinicopathological factors of ESCC patients with radiotherapy

Clinicopathological factors	Number of patients	%
Age(years)[range 39-74; median 60]		
≤60	3	13.0
>60	20	87.0
Gender		
Male	21	91.3
Female	2	8.7
Tumor location		
Upper	8	34.8
Middle	6	26.1
Lower	9	39.1
cN		
N0	4	17.4
N1	9	39.1
N2	9	39.1
N3	1	4.4
cT		
T2	6	26.1
T3	11	47.8
T4	6	26.1
cTNM stage		
II	1	4.3
III	10	43.5
IV	12	52.2
Tumor differentiation		
Well	1	4.3
Modest	10	43.5
Poor	12	52.2
Radiotherapy		
TD 61.2 Gy/34 F	15	65.2
Others	8	34.8
Chemotherapy		
Paclitaxel + Platinum	21	91.3
Others	2	8.7
